# Functional Diversity Responses to Temperature Vary Spatially and With Protection Status in Wintering Waterbird Assemblages

**DOI:** 10.1111/ele.70467

**Published:** 2026-10-20

**Authors:** Tanith B. Hackney, Hannah S. Wauchope, Pol Capdevila, Christopher F. Clements

**Affiliations:** ^1^ School of Biological Sciences University of Bristol Bristol UK; ^2^ School of GeoSciences The University of Edinburgh Edinburgh UK; ^3^ Departament de Biologia Evolutiva, Ecologia i Ciències Ambientals, Facultat de Biologia Universitat de Barcelona (UB) Barcelona Spain; ^4^ Institut de Recerca de la Biodiversitat (IRBio), Universitat de Barcelona (UB) Barcelona Spain

**Keywords:** Bayesian models, climate change, conservation, International Waterbird Census, Ramsar convention, redundancy, resilience, Special Protection Area

## Abstract

Wetlands support disproportionately high biodiversity, including waterbirds, which underpin key ecological functions and regulatory services. This has led to efforts to protect these crucial sites, but whether these protections can maintain waterbird assemblages in the face of new threats such as climate change is poorly understood. Here, we calculated functional redundancy and three metrics of functional diversity from over 45,000 waterbird censuses, spanning four decades of rising winter temperatures for 2792 protected and unprotected sites in Europe. We modelled the effect of average winter temperature and the trend over time for each of these metrics. Our results suggest functional diversity generally increased in warmer winters and over time, but redundancy declined, and the effect of temperature varied spatially and between levels of protection. This suggests abundance and distribution shifts due to climate change are altering functional diversity and resilience of waterbird assemblages both inside and outside protected areas across Europe.

## Graphical abstract

Using > 45,000 waterbird censuses at 2792 European sites, we found functional diversity has increased over time and in warmer winters, but functional redundancy has declined. Ramsar and Special Protection Area sites had the greatest increase in functional richness but the greatest decline in functional redundancy in warmer winters, especially further north and east. This study suggests rising temperatures could alter the functional diversity and resilience of waterbird assemblages across Europe.

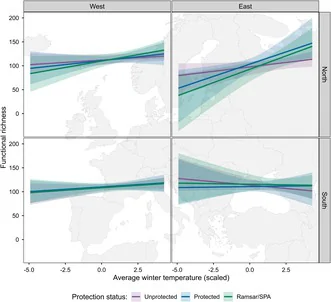

## 1 Introduction

Wetlands have been lost and degraded faster than any other ecosystem (Davidson 2014; Millennium Ecosystem Assessment 2005). Europe alone has lost over half its wetlands, with some European countries losing > 75% since 1700 (Fluet‐Chouinard et al. 2023). This is concerning because wetlands provide numerous ecosystem services, for example carbon sequestration, water purification, flood mitigation, and cultural services such as recreation or tourism (Costanza et al. 2014; Convention on Wetlands 2021; Mitsch et al. 2015), and support disproportionately high biodiversity (Tickner et al. 2020). A main threat is climate change (Desta et al. 2012; Salimi et al. 2021; Xi et al. 2021), which causes changes in species' phenology, physiology, life history and ranges, affecting species interactions and community composition (Bellard et al. 2012). Climate change‐induced biodiversity loss can alter ecosystem functioning (through the loss of unique species characteristics—their functional traits) and undermine resilience to other threats (Isbell et al. 2023; Oliver, Isaac, et al. 2015).

The contribution of communities to ecosystem functioning can be measured through functional diversity (Mammola et al. 2021). Functional diversity describes the variation in traits within a community influencing ecosystem functioning and consists of three primary components: functional richness is the amount of trait space occupied by species, whereas functional evenness and divergence describe the arrangement of species and their abundances within functional trait space (Mason et al. 2005; Carmona et al. 2016). Communities with higher functional diversity also tend to be more resilient, as they have a higher variation in potential responses to disturbances (Laliberté et al. 2010; Mori et al. 2013; Sasaki et al. 2019; White et al. 2023) or can better resist invasion (Carturan et al. 2022; Cheng et al. 2024; Levine et al. 2004). Additionally, high functional redundancy, that is, communities in which multiple species share similar functional traits, means surviving species can compensate for species loss by maintaining ecosystem functions (Mori et al. 2013; Naeem 1998). Therefore, protecting wetland biodiversity is essential for maintaining ecosystem functioning and the adaptive capacity of wetlands (Jacob et al. 2025). However, rising temperatures are causing shifts in ecological communities and species' distributions (Pearce‐Higgins et al. 2015; Pecl et al. 2017), which increasingly threatens functional diversity (Scherer et al. 2023; Stewart et al. 2022).

Waterbirds are a key group driving function and regulatory services in wetlands (Green and Elmberg 2014). For example, many waterbirds maintain species diversity through the spread of animal and plant propagules (Green et al. 2023) or herbivory that enhances plant diversity and primary productivity (Cargill and Jefferies 1984; Nolet 2004). Waterbirds are also important for nutrient cycling and trophic regulation (Boros et al. 2025; Chen et al. 2023; Mukherjee et al. 2024) among many other functions. However, species responses to climate change can also differ based on their traits, such as body size or feeding behaviour (Feng et al. 2025; Pavón‐Jordán et al. 2019; Ramírez et al. 2018). For example, deep‐water feeding and shallow‐water feeding waterbird species shift their population distributions differently in response to winter weather conditions (Pavón‐Jordán et al. 2019; Ramírez et al. 2018). Also, large species withstand colder temperatures but show fewer occurrence shifts compared to smaller, more warm‐adapted species (Dalby et al. 2013; Marchowski et al. 2017). As a result, many wintering waterbird population abundances have increased in the north but declined in the south in response to climate change (Amano et al. 2020), which is reflected by a northeastward shift in wintering distributions (Lehikoinen et al. 2013; Maclean et al. 2008; Musilová et al. 2018; Ramo et al. 2015). With increasingly warm winters in Europe (Twardosz et al. 2021), rising temperatures may affect the diversity of functional traits in wintering waterbird assemblages, which could impact ecosystem functioning.

Protected areas may help mitigate negative functional diversity changes because these waterbird assemblages should be better able to cope with and adjust to climate change (Gaget, Johnston, et al. 2021; Gaget, Pavón‐Jordán, et al. 2021; Gaüzère et al. 2016). Managing local wetland sites to reduce local stressors can build resilience to larger‐scale threats like climate change (Green et al. 2017; Scheffer et al. 2015). In addition, protected areas that are managed specifically for wetlands, for example through the Ramsar Convention (1971), on average have a more positive impact on populations than other protected areas (Wauchope et al. 2022). In Europe, Gaget et al. (2018) found that sites where both the Birds Directive and the Bern Convention, two important conservation policies, were enforced, warm‐dwelling species increased in abundance whilst cold‐dwelling species remained stable, suggesting these communities were better able to adjust to temperature change. However, the effect of protected areas on functional diversity, and their ability to buffer communities against negative changes, remains unstudied.

Here we apply Bayesian multi‐level models to a large dataset of waterbird abundance counts at protected and unprotected sites across Europe. We ask whether rising winter temperatures are associated with changes in functional diversity (measured through richness, evenness, and divergence) and functional redundancy. We consider change in two ways: first whether diversity metrics are related to winter temperatures, assessing the immediate ability of waterbirds to adjust where they winter based on local conditions. Second, whether there is a consistent trend through time, assessing longer term shifts in waterbird assemblages. We hypothesise that functional diversity and redundancy will be higher in warmer winters in the north and northeast, but lower in the south. We expect this will be mirrored in temporal trends, with increases in the north but declines in the south. However, we expect waterbird assemblages in protected areas, especially those designated and managed for waterbirds, to maintain functional diversity through time and with rising temperatures, with increases in the north and east, thereby indicating an enhanced ability to adapt and respond to temperature change.

## 2 Material and Methods

All data cleaning and analyses were carried out using the statistical programming language *R* version 4.5.1 (R Core Team 2025).

### 2.1 Waterbird Count Data

The International Waterbird Census (IWC) is a global monitoring programme consisting of annual counts of waterbirds at wetland sites in over 100 countries, coordinated by Wetlands International since 1967 (Wetlands International 2018). The IWC includes 34 bird families, where it defines a waterbird as a bird species that is ‘ecologically dependent on wetlands’. The standardisation of this census–counting the same sites in the same way every year with expert counters–allows the calculation of population trends through time. Most of the counts occur in January, which is typically midwinter in the Northern Hemisphere, therefore representing wintering waterbird populations (Wetlands International 2018). We used the same dataset as in Wauchope et al. (2022) but filtered the data to include only European countries with time series spanning at least five years, and with a maximum of one‐year time gaps to reliably identify trends (Wauchope et al. 2019; White 2019). We also cut time series to start from 1980 onwards to ensure unprotected sites were comparable with protected sites under the Ramsar Convention which were first designated in the 1970s (see Section 2.2 below). For gaps, where a species had not been recorded in a given year and site but other species had, zeroes were imputed, as this suggests the species was absent in that year, as described in Wauchope et al. (2022). To choose sites that were ecologically relevant and to allow us to calculate functional diversity metrics, we selected sites where at least ten waterbird species had been recorded throughout the time series, and where there were always at least two species present in any given year. This resulted in 2792 sites across 37 countries and 213 species within 17 families between 1980 and 2018 across Europe (Figure 1). The mean time series length was 17 years, and the majority of the timeseries had no missing years (see Supplementary Methods for full data description).

**FIGURE 1:**
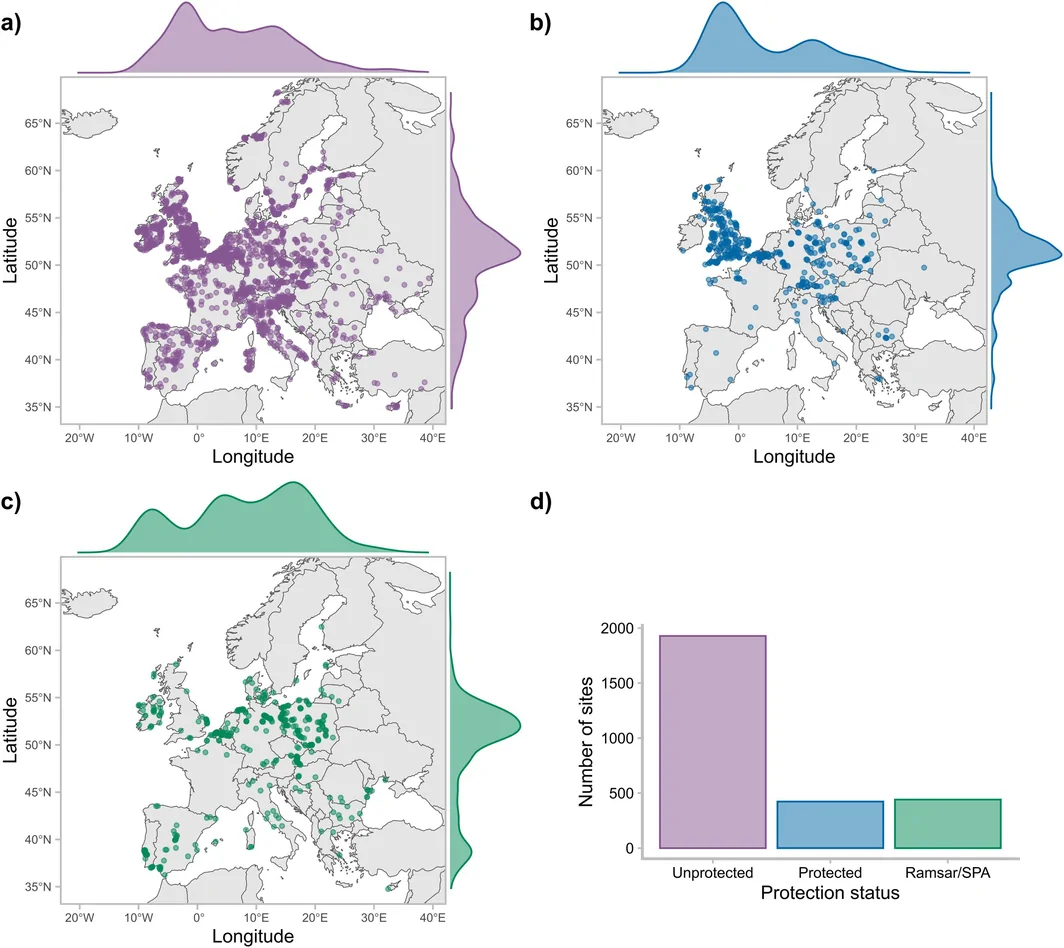
Distribution of International Waterbird Census sites across Europe and their protection status: (a) unprotected areas (purple, 1928 sites); (b) non‐targeted protected areas (blue, 423 sites); and (c) Ramsar Convention and Special Protection Areas (green, 441 sites). Density plots represent the distribution of sites across latitude and longitude, and (d) is the number of sites in each protection status.

### 2.2 Protected Area Data

The Ramsar Convention on Wetlands (‘Ramsar’) and the Special Protection Areas under the Birds Directive (‘SPA’) classify internationally important wetlands as those holding more than 1% of a waterbird population or at least 20,000 waterbirds (Wetlands International 2018). As a proxy for protection level, we considered sites designated as Ramsar or a SPA to have the highest protection because these sites tend to have active management and protection in place for waterbirds (Wauchope et al. 2022). We assumed all other protected sites were not specifically managed for waterbirds, with more general, ‘non‐targeted’ management in place.

We downloaded global protected area data from the World Database on Protected Areas (UNEP‐WCMC and IUCN 2024). We removed protected areas where the status was proposed or had not been reported, where the year of designation was not recorded, or any that were designated as a United Nations Educational, Scientific, and Cultural Organization (UNESCO) Biosphere Reserve because ecosystems are often not the priority in such areas (Coetzer et al. 2014). With the remaining protected areas, we assigned each to a site if it overlapped with a circular 250‐m radius around each site centroid to account for spillover effects or inaccuracies in coordinates. Our analyses required us to assign just one protected area designation to each site; however, some sites were overlapped by multiple protected areas. Here, we prioritised keeping Ramsar or SPA designations, followed by the earliest designation year. We selected only sites that had either been fully protected or unprotected for all the years of the time series to avoid changes in protection affecting the calculated metrics. We defined each site as ‘unprotected’, ‘non‐targeted protected’, or ‘Ramsar/SPA’.

### 2.3 Climate Data

Climate change likely impacts waterbird winter distributions more than their breeding distributions because they respond more rapidly to changes in climatic and environmental conditions during winter (Clausen and Madsen 2016; Guillemain et al. 2013; Knudsen et al. 2011). Therefore, we expect that local winter temperature will determine the diversity of species and functions present at wintering sites (Knudsen et al. 2011). Consequently, we downloaded monthly average air temperature at two metres (‘tas’) for each site's coordinates for the years 1979–2018 from the open access database CHELSA v2.1 (Brun et al. 2022; Karger et al. 2017). These data have a high resolution of 30 arcseconds (~1 km^2^). For each site, we calculated the average temperature across the winter months, which we considered to be the November and December of the previous year and the January of the year in which the count took place (most counts occur in January). Units were given in Kelvin × 10, so we converted to Celsius using the formula:

Celsius=0.1×tas−273.15

### 2.4 Trait Data

We compiled a list of traits important for ecosystem functioning or helpful in determining responses to environmental change (Luck et al. 2012). Avian morphological traits accurately predict categorical traits such as trophic niche (Pigot et al. 2020), trophic level (Dehling et al. 2016), and primary lifestyle and habitat (Tobias et al. 2022). Avian categorical traits such as foraging niche or diet can also be subjective and lacking in detail, so their use has been questioned, particularly given the availability of detailed morphological trait data (Kohli and Jarzyna 2021; Tobias et al. 2022). Consequently, we chose eight continuous traits that also had available data with excellent species coverage (Table 1) and that were not highly correlated. For the full justification for each trait see Supplementary Methods.

**TABLE 1:** Traits selected for calculating functional diversity indices, alongside their description and data source: AVONET (Tobias et al. 2022); EltonTraits (Wilman et al. 2014); or Amniote (Myhrvold et al. 2015).

Trait category	Trait	Description	Source	Species coverage
Bill morphology	Bill length	Length of bill from nares to tip (cm)	AVONET	100%
	Bill width	Width of bill (cm)	AVONET	100%
Wing morphology	Hand‐wing index	Ratio of wing length and secondary feather length (cm)	AVONET	100%
Body size	Body mass	Weight (g)	AVONET	100%
	Tarsus length	Length of tarsus (cm)	AVONET	100%
Diet	Diet breadth	Number of diet types	EltonTraits	100%
Life history	Clutch size	Average number of eggs per clutch	Amniote	99.6%
	Maximum longevity	Maximum recorded lifespan (years)	Amniote	80.5%

We were able to obtain data for every species for all traits except longevity and clutch size. The Pygmy Cormorant (*Microcarbo pygmaeus*) was the only species missing clutch size, so the average clutch size was taken from Barati et al. (2008). 19.5% of species did not have data on maximum longevity. After testing multiple methods, we imputed these missing trait values using *Rphylopars* version 0.3.10 (Goolsby et al. 2024), including traits and phylogeny (see Supplementary Methods). We used the BirdLife International taxonomy, derived from the Handbook of the Birds of World and BirdLife International Illustrated Checklist of the Birds of the World (2024). This is the same taxonomy used in the AVONET database. EltonTraits and Amniote use the taxonomy from Jetz et al. (2012). Synonyms in EltonTraits were manually identified and we used these to match any species in EltonTraits and Amniote databases to AVONET.

### 2.5 Functional Diversity

To characterise the functional diversity of each bird assemblage and time point, we built a multidimensional functional space by performing a principal component analysis (PCA) on the scaled functional traits of each site and year using the R function *prcomp*. To estimate the patterns of organisation of species in the functional space, we used the trait probability density (TPD) (Carmona et al. 2016), estimated using the *TPD* package (Carmona et al. 2019). TPD computes species' probabilistic distribution in the functional space using multivariate kernel density estimation (Carmona et al. 2016), thus reflecting the density of species in that particular area of the functional space (Carmona et al. 2019). We estimated the kernel density estimation for each species as a multivariate normal distribution centred at the species' coordinates in the PCA space (Carmona et al. 2016, 2019). Standard deviation was computed using the unconstrained bandwidth selectors from the *Hpi.diag* function in the *ks* package (Duong 2024). To build the TPD we used the first three principal components, which together accounted for 73.4% of variance in waterbird functional traits.

To characterise functional diversity, we estimated three primary components; functional richness, functional evenness, and functional divergence (Mason et al. 2005; Villéger et al. 2008). Functional richness describes how much of functional trait space is covered by species, formally calculated as the sum of the hypervolumes in which the TPD is greater than zero (Carmona et al. 2016). It only considers presence or absence of species, not their abundances. Functional evenness and divergence describe the arrangement of species and their abundances within functional trait space. Evenness measures how evenly abundance is distributed among ecological units (e.g., traits or species). Divergence describes how abundance is spread, either towards the extremes or centre of a functional volume. These three components were estimated using the *REND* function in the *TPD* package (Carmona et al. 2019).

We also estimated functional redundancy, which is not a component of functional diversity but reflects how many species on average are present in each cell of the functional space. Functional redundancy is thus an indicator of the resilience of the community to species loss (Oliver, Heard, et al. 2015), with high redundancy being able to buffer species loss. Here, we used the function *redundancy* in the *TPD* package (Carmona et al. 2019) to calculate relative redundancy, a measure of functional redundancy that accounts for species richness (calculated as functional redundancy divided by species richness minus one).

### 2.6 Statistical Analysis

To model the effect of winter temperature on functional diversity and redundancy, and their temporal trends, we fit a set of Bayesian multilevel models with either functional richness, divergence, evenness, or redundancy as the response variable. We fit two sets of models, testing the effect of either (1) temperature or (2) time on these responses, resulting in eight models overall. In all cases, we also examined whether relationships differed through space or according to protection status.

To quantify instantaneous effects of winter temperature, we modelled interactive fixed effects of average winter temperature, latitude, longitude, and protection status (‘unprotected’, ‘non‐targeted protected’, and ‘Ramsar/SPA’). To account for site‐ or year‐specific variation, we included random intercepts for site and year. To understand whether responses to winter temperature may translate to temporal trends and followed the same spatial pattern, we modelled interactive fixed effects of year, latitude, longitude, and protection status, with a random intercept and slope for site.

In both sets of models, the continuous fixed effects (latitude and longitude, temperature or year) were scaled or centred to facilitate model convergence. The time series for each site was modelled with an autoregressive‐1 temporal autocorrelation structure to account for correlation between functional diversity in neighbouring years. For functional divergence, evenness, and relative redundancy, which are bounded between 0 and 1, we used weakly informed priors with an ordered beta regression distribution. For functional richness neither a lognormal nor gamma prior distribution fit our data well so—given that we are not predicting outside the range of our data—we chose a prior Gaussian distribution for functional richness which fit the data with a good approximation. All model formulae and priors are in the Supplementary Methods.

Models were run for 8000 iterations, with a warmup of 4000 iterations. Distributions and plots were based on 1000 random draws from the posterior distribution. All models were fit with the *ordbetareg* package version 0.8 (Kubinec 2023) and *brms* package version 2.22.0 (Bürkner 2017) using the *cmdstanr* version 0.9.0 backend (Gabry et al. 2025) in *R*. We evaluated effects using the 95% credible intervals, with an overlap with zero indicating no effect. We assessed model fit using visual inspection of trace plots, Rhat values, and posterior predictive checks (see Model Diagnostics in the Supporting Information).

## 3 Results

### 3.1 Correlation With Winter Temperature

Functional richness was higher in warmer winters (Figure 2; Table S5). There was a negative interaction between temperature and longitude, but a positive interaction between temperature, longitude, and latitude, suggesting the rise in functional richness was less positive overall in the east, but most positive in the northeast in warmer winters. However, functional richness was generally higher in warmer winters than cold winters further east in non‐targeted protected sites compared to unprotected sites. The rise in functional richness in warmer winters was particularly apparent in Ramsar/SPA sites. There was also a positive interaction between temperature, latitude, and Ramsar/SPA status suggesting that this was most positive further north. Functional divergence was also higher in warmer winters further north (Figure S9; Table S6), whereas functional evenness increased most in warmer winters further south and east (Figure S10; Table S7). Protection status had no effect on the response of divergence or evenness to winter temperature. Relative redundancy (functional redundancy accounting for species richness–see Section 2) was lower in warmer winters, with eastern Ramsar/SPA sites experiencing the strongest decline, compared to unprotected sites (Figure 3; Table S8). However, relative redundancy increased with temperature in non‐targeted protected sites in northeastern Europe.

**FIGURE 2:**
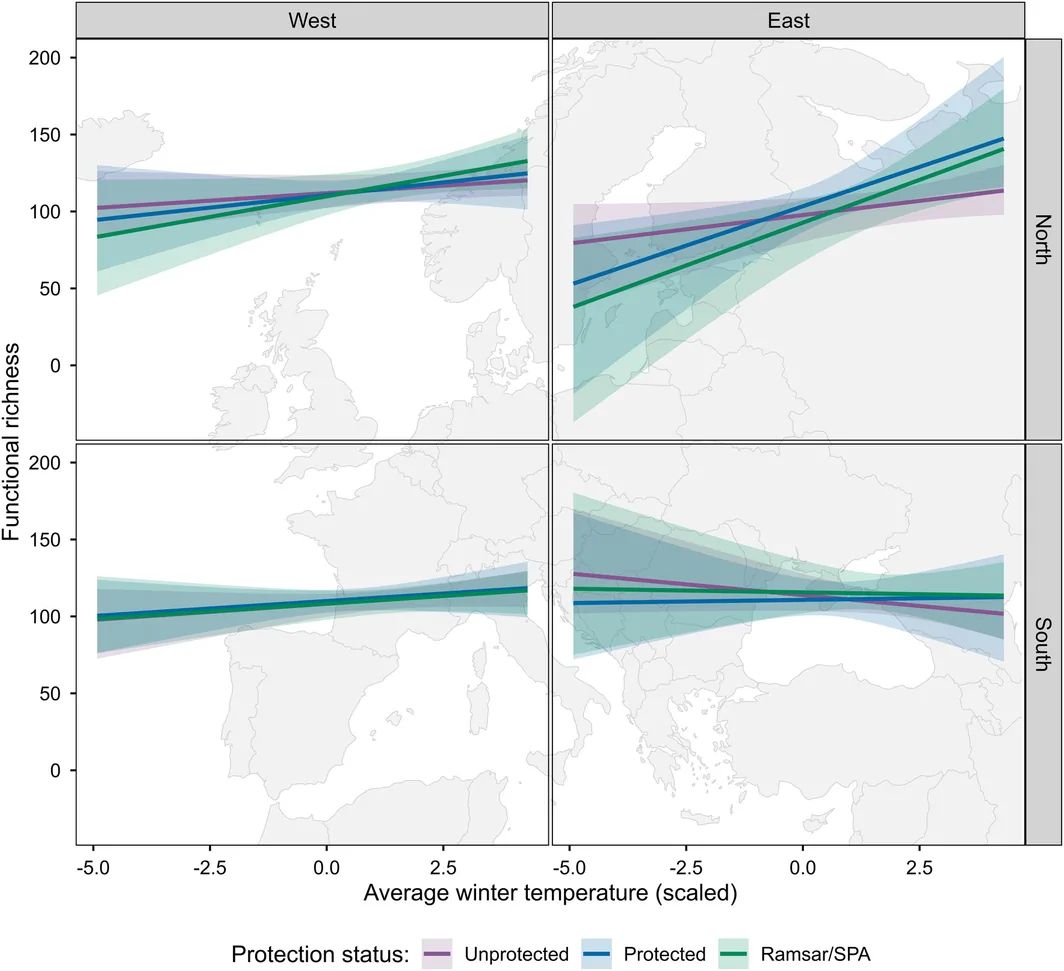
Posterior predictions of functional richness with scaled average winter temperature from 1000 random draws from the model, with the ribbons representing the 95% credible intervals. The part of Europe behind each facet grid square is the area the trend line represents, predicted from 25 longitude and latitude values on either side of the mid latitude and longitude.

**FIGURE 3:**
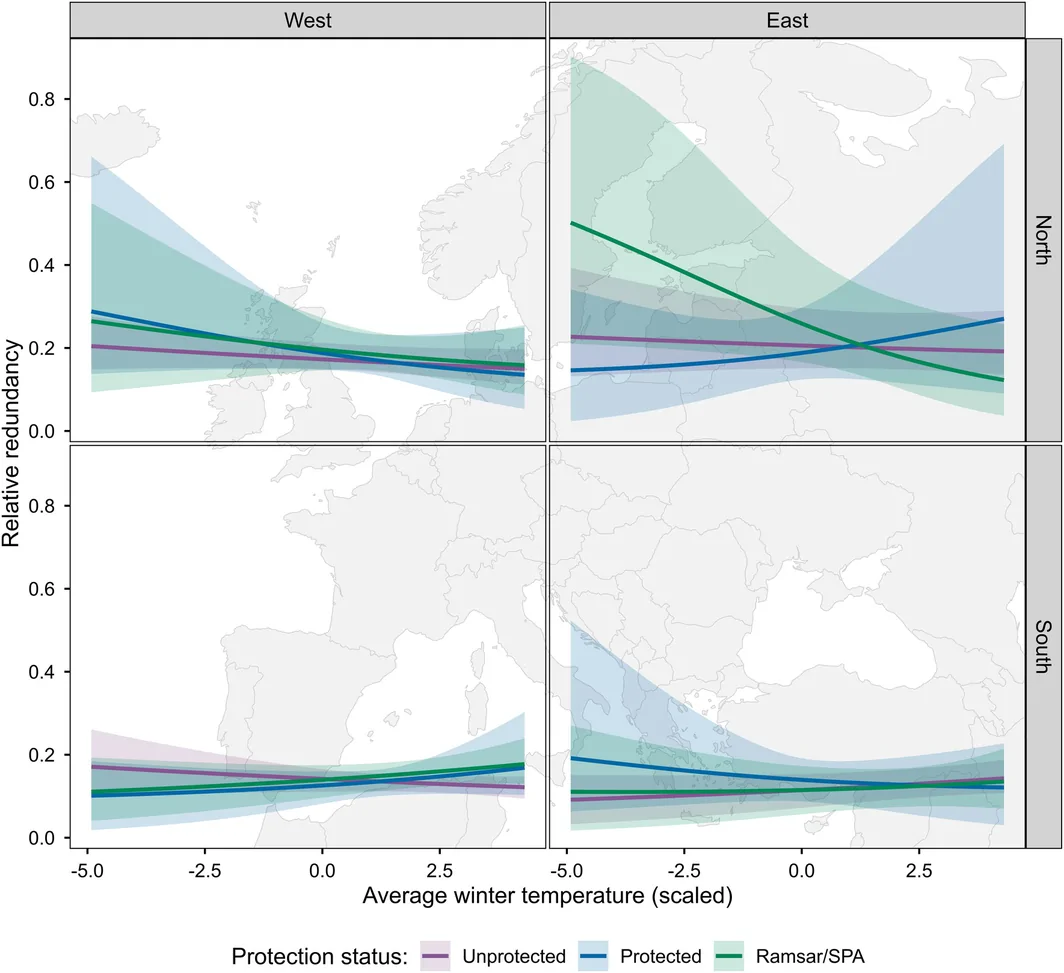
Posterior predictions of relative redundancy with scaled average winter temperature from 1000 random draws from the model, with the ribbons representing the 95% credible intervals. The part of Europe behind each facet grid square is the area the trend line represents, predicted from 25 longitude and latitude values on either side of the mid latitude and longitude.

### 3.2 Trends Over Time

Overall, functional richness increased over the study period (Figure 4; Table S9). However, this trend was less positive further north and with Ramsar/SPA status. There was no effect of longitude on these interactions. Functional divergence increased over the study period in unprotected and non‐targeted protected sites, especially further north, but declined in Ramsar/SPA sites (Figure 5; Table S10). Functional evenness had no clear trends over time (Figure S11; Table S11). Relative redundancy declined over time across all protection statuses and latitudes, but less so further east (Figure S12; Table S12).

**FIGURE 4:**
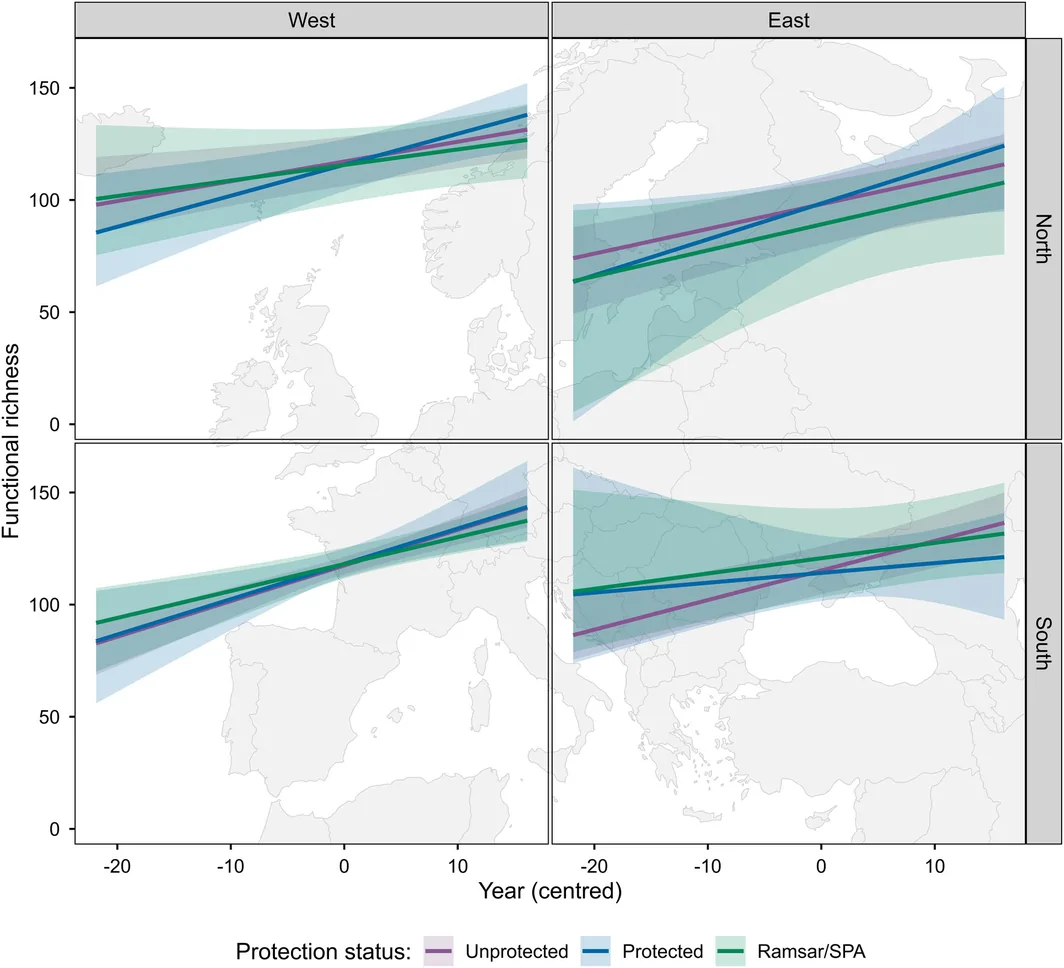
Posterior predictions of functional richness over time from 1000 random draws from the model, with the ribbons representing the 95% credible intervals. The part of Europe behind each facet grid square is the area the trend line represents, predicted from 25 longitude and latitude values on either side of the mid latitude and longitude.

**FIGURE 5:**
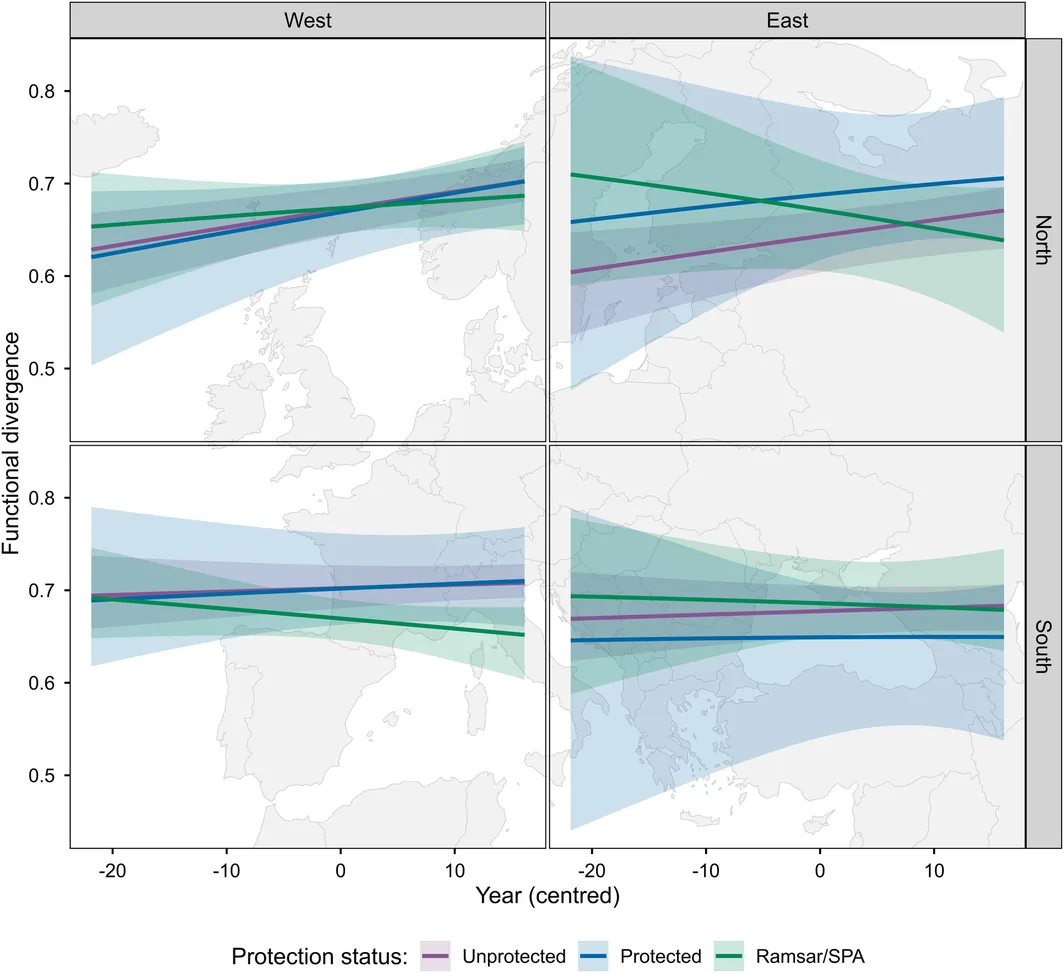
Posterior predictions of functional divergence over time from 1000 random draws from the model, with the ribbons representing the 95% credible intervals. The part of Europe behind each facet grid square is the area the trend line represents, predicted from 25 longitude and latitude values on either side of the mid latitude and longitude.

## 4 Discussion

Using a dataset of 71,286 waterbird population trends across 2792 sites and 37 countries, we show that functional diversity has increased over the last four decades, and in warmer winters, across protected and unprotected sites in Europe. However, the strength of these responses varied spatially, and, unlike functional diversity, redundancy declined over time and in warmer winters. Previous studies have assessed waterbird functional diversity using a few functional groups or only in small regions or a single country (de Arruda Almeida et al. 2019; Özgencil et al. 2020; Santos‐Tovar et al. 2024). To our knowledge, this is the first study to assess trends in waterbird functional diversity and redundancy at a continental scale anywhere in the world.

Traits mediate species responses to climate change (Bourhis et al. 2025; Luck et al. 2012), and some waterbird species are more likely to benefit from warmer winters in the north, such as open water feeders and smaller‐bodied species (Dalby et al. 2013; Pavón‐Jordán et al. 2019). This could explain why functional divergence increased over time and in warmer winters, especially at high latitudes. Increasing divergence suggests species abundances at the extremes of the functional space are increasing compared to those close to the centre, resulting in greater niche differentiation (Mouchet et al. 2010). Functional evenness also increased in warmer winters. This could reflect simultaneous declines in formerly abundant species and increases in novel species, leading to greater regularity in abundance across the trait space in warm winters (Barnagaud et al. 2017). Whereas in colder winters, functional evenness may be lower because parts of the functional space are empty, or because in colder winters a few cold‐tolerant species dominate, whilst others are at lower abundance (Mouchet et al. 2010). Wetland sites in eastern Europe are becoming the most climatically suitable for many species with climate change (Breiner et al. 2022), possibly explaining the increase in evenness in the east.

We expected that waterbirds would winter further north and northeast when conditions are suitable–either to avoid high temperatures in the south, or to reduce energetic costs by remaining closer to their breeding grounds (Nuijten et al. 2020; Ramo et al. 2015; Rehfisch et al. 2004; Reneerkens et al. 2020)–resulting in higher functional richness in warmer winters in the north. A polewards shift with rising temperatures has been observed in many other taxa (Hickling et al. 2005; Mason et al. 2015; Ramalho et al. 2023; Rubenstein et al. 2023; Zurell et al. 2024). In line with this, we found functional richness correlated positively with local average winter temperature, and this relationship was particularly strong in northeastern Europe. Functional richness also increased over time, which we expected with rising winter temperatures. However, contrary to our expectations, the positive temporal trend was strongest in the south. Functional richness is influenced by species at the extremes of trait space (Mouchet et al. 2010; Villéger et al. 2008) and sensitive to species richness (de Bello et al. 2021). African and Middle Eastern wetland sites' suitability for waterbirds is declining the most with climate change (Breiner et al. 2022), so the steeper temporal trend in the south may be explained by Southern Hemisphere species with novel functions shifting their distributions northwards to southern Europe with rising temperatures (Bates et al. 2014; Burrows et al. 2011; Princé and Zuckerberg 2015), rather than the local temperature at the new site. Similar trends have been observed in Spain, where species and functional richness were highly correlated, suggesting that the increase in functional richness is driven by the arrival of new species and therefore functions (de Arruda Almeida et al. 2019). Further research is needed to understand the drivers of these changes, for example whether resident species range shifts or migrant wintering site shifts, or both, could explain the changes in functional diversity. Additionally, different waterbird families could be responding differently to climate change, helping to explain these changes, or highlight families that are losing or gaining functions.

A higher diversity of functional traits could have positive effects on ecosystem function and on the services provided by waterbirds and wetlands. The rise in functional richness could introduce new functions to these wetlands, filling more niche space (de Bello et al. 2021; Mori et al. 2013), which could improve ecosystem functioning (Díaz and Cabido 2001; Gagic et al. 2015) and resilience (Oliver, Heard, et al. 2015; Yachi and Loreau 1999). Higher functional diversity also buffers ecosystems to disturbance, leading to greater stability (Cadotte et al. 2011; Walker et al. 1999). However, these increases could also reflect shifting baselines, with recovery following historic biodiversity loss (Soga and Gaston 2018), due to greater wetland protection and restoration efforts across Europe (Verhoeven 2014).

Functional richness increased the most in Ramsar/SPA sites in warmer winters. This suggests that waterbirds may preferentially choose managed protected sites in warmer winters, supporting the idea that these protected areas may help species respond to or cope with climate change (Gaget, Pavón‐Jordán, et al. 2021; Gaget et al. 2025). However, we found no effect of protection on responses of functional divergence or evenness to warmer winters. Additionally, Ramsar/SPA sites had a slower temporal increase in functional richness compared to unprotected sites. Functional divergence in Ramsar/SPA sites declined over time, suggesting that species with novel functions are likely to be at low abundance in these sites, with trait abundance becoming more clustered at the centre of the functional space. Therefore, even though richness still increased, these species with novel functions may not have much impact on functioning if their abundance is low (Díaz and Cabido 2001).

Functional richness also increased in unprotected sites, both in warmer winters and over time. As distributions change, unprotected and protected areas lacking waterbird‐specific management may become increasingly important wintering and staging areas (Lehikoinen et al. 2013; Nagy et al. 2022; Musilová et al. 2015; Pavón‐Jordán et al. 2015, 2020), enhancing their functional diversity. Pavón‐Jordán et al. (2015) found that although smew ( *Mergellus albellus* ) abundance has increased more in SPAs in the northeastern and southwestern range, there are still gaps in protection with many important wintering areas unprotected. We found a positive interaction between non‐targeted protected areas and longitude on functional richness, likely due to more species overwintering in these areas as they became more climatically suitable (Breiner et al. 2022; Gaget et al. 2025; Lehikoinen et al. 2019; Thomas et al. 2012). Limited protection in newly suitable sites may threaten populations in the future because, without adequate protection and management, these waterbird assemblages could become increasingly vulnerable to other threats. For example, greater scaup ( *Aythya marila* ) have shifted their distribution northeastwards in Europe, exposing them more to threats such as bycatch mortality and food scarcity (Marchowski et al. 2020). Protecting species and functional diversity in the future will require designating new areas that consider the effects of climate change (Guillemain and Hearn 2017; Nagy et al. 2022).

Even though waterbird assemblages have become more functionally diverse, these new assemblages could be more vulnerable to future threats (Fonesca and Ganade 2001). For example, a decline in redundancy can increase functional divergence and evenness (Mouillot et al. 2013). Indeed, redundancy was generally lower in warmer winters, especially towards the west, and redundancy declined over time across all Europe, suggesting there is less overlap in species' trait space. This could be driven by increased competition (Pimiento et al. 2020), and potentially undermines the resilience of these assemblages (Naeem 1998; Biggs et al. 2020). The simultaneous rise in functional richness and decline in relative redundancy suggests that the changes observed in these assemblages is due to niche expansion, where additional species occupy new areas of niche space, rather than niche packing, where species occupy similar or more specialised areas of the same niche space (Pagani‐Núñez et al. 2019; Pigot et al. 2016). Although non‐targeted protected areas had a slight increase in redundancy in northeast Europe, redundancy in further eastern Ramsar/SPA sites declined rapidly with warmer winters compared to the rest of Europe. This may be an early sign of future vulnerability in Ramsar/SPA sites.

Although local‐scale winter temperature appears to be important, it cannot explain all temporal trends. Larger‐scale temperature changes, or other factors such as land‐use change, may influence these trends, which we could not capture in this study. We were also unable to account for wetland characteristics such as size or isolation directly, but these are somewhat accounted for within the site random effect in our models. This accounts for stable site‐level variation, and we found that it does not drive directional trends in our results, indicating that our results are not confounded by differences in these variables (see Supporting Information). However, the random effect captures greater variation than our fixed predictors, suggesting wetland characteristics may contribute to site‐level differences in responses. Further research is needed to understand how these characteristics might influence the strength of responses at the site level and interact with other threats, which could further direct protection efforts.

Migratory waterbirds are also impacted by phenomena outside their wintering grounds (Almaraz et al. 2012; Webster and Marra 2005) and only considering local winter conditions may miss important information (de Arruda Almeida et al. 2019). Although globally functional richness for many taxa is declining (Brodie et al. 2021; Carmona et al. 2021; Scherer et al. 2023), regional or local increases may still occur as species ranges shift in response to climate change (Antão et al. 2020; Scherer et al. 2023), and temperate regions may act as ‘sinks’ for tropical or migratory species that are more vulnerable to climate change (Deutsch et al. 2008; Matuoka et al. 2020). This could be why functional richness has increased in southern Europe. However, range expansion often outpaces local extinction, creating a temporary rise in diversity (Antão et al. 2020; Bates et al. 2014; Dullinger et al. 2012; Poloczanska et al. 2013; Scherer et al. 2023). These increases may not persist if temperatures continue to rise beyond their thermal limit (Deutsch et al. 2008). Responses may also differ depending on threat identity and intensity, for example intermediate disturbance levels can increase competition, leading to greater functional diversity (Oliveira and dos Anjos 2023), which could also be true for warming. Moreover, rare species are particularly vulnerable (Finderup Nielsen et al. 2019; Mouillot et al. 2013), with declining redundancy meaning these communities are less able to buffer against species loss. Changing species composition and functional diversity can also lead to novel biotic interactions (Gilman et al. 2010; Parmesan 2006) resulting in ecosystem‐level changes (Pecl et al. 2017).

Overall, our results suggest that the previously documented changes in waterbird abundance and occurrence (Amano et al. 2020; de Arruda Almeida et al. 2019; Gaget et al. 2025; Lehikoinen et al. 2013; Pavón‐Jordán et al. 2020) have resulted in major changes in functional diversity in Europe. In warmer winters, functional richness and evenness increase across all latitudes, and divergence at higher latitudes. Given their high complementarity (Gagic et al. 2015; Villéger et al. 2008), this suggests that assemblages are more functionally diverse in warmer winters, especially further northeast. However, redundancy declined, suggesting these assemblages may be less resilient. It also appears that waterbird assemblages in Ramsar and Special Protection Area sites are responding differently compared to other sites, although we do not attempt to make any causal claims on the effect of protection as we were unable to produce a satisfactory causal model (Geldmann et al. 2025; Wauchope et al. 2022). However, our study provides the first Europe‐wide evidence that waterbird assemblages have undergone changes in functional diversity and redundancy, with important implications for ecosystem functioning and resilience under climate change.

## Author Contributions

T.B.H., H.S.W., P.C. and C.F.C. conceived and designed the study. T.B.H. and H.S.W. cleaned and collated the data for the analysis. T.B.H. analysed the data and wrote the first draft. T.B.H., H.S.W., P.C. and C.F.C. all contributed to writing and editing the manuscript. All authors gave their approval before publication.

## Funding

This work was supported by the Natural Environment Research Council.

## Supporting information


**Table S1:** All 213 species in the final dataset used for the functional diversity calculations for the models. There were 250 species used for trait imputation, but we lost 37 species during the data cleaning steps after this, resulting in 213 species.
**Table S2:** Variance inflation factors (VIF) with 95% confidence for each of the models with temperature as a predictor. All have low correlation.
**Table S3:** Variance inflation factors (VIF) with 95% confidence for each of the models with time as a predictor. All have low correlation.
**Table S4:** Bayesian *R* ^2^ with 95% credible intervals for each of the models with temperature or time as a predictor, and functional richness, divergence, evenness, or redundancy as the response variable.
**Table S5:** The coefficients from the functional richness and temperature model, including the median and lower and upper 95% credible intervals of the posterior distribution. Rhat close to one indicates convergence, and ESS is the effective sample size.
**Table S6:** The coefficients from the functional divergence and temperature model, including the median and lower and upper 95% credible intervals of the posterior distribution. Rhat close to one indicates convergence, and ESS is the effective sample size.
**Table S7:** The coefficients from the functional evenness and temperature model, including the median and lower and upper 95% credible intervals of the posterior distribution. Rhat close to one indicates convergence, and ESS is the effective sample size.
**Table S8:** The coefficients from the relative redundancy and temperature model, including the median and lower and upper 95% credible intervals of the posterior distribution. Rhat close to one indicates convergence, and ESS is the effective sample size.
**Table S9:** The coefficients from the functional richness and time model, including the median and lower and upper 95% credible intervals of the posterior distribution. Rhat close to one indicates convergence, and ESS is the effective sample size.
**Table S10:** The coefficients from the functional divergence and time model, including the median and lower and upper 95% credible intervals of the posterior distribution. Rhat close to one indicates convergence, and ESS is the effective sample size.
**Table S11:** The coefficients from the functional evenness and time model, including the median and lower and upper 95% credible intervals of the posterior distribution. Rhat close to one indicates convergence, and ESS is the effective sample size.
**Figure S1:** (a) The frequency of year in which the time series starts. The highest start year was 1980 but there is good representation across all years with peaks in 1995 and 2011. (b) Number of sites in each year. (c) The length of time series. Most time series lengths are well represented in the dataset. (d) Proportion of missing years (gaps) in time series. Most time series had no gaps.
**Figure S2:** Comparison of maximum longevity distributions for each imputation method, *Rphylopars* and *missForest*, with and without traits and phylogeny. (a) shows the frequency distributions for all maximum longevity values for *missForest* and *Rphylopars*, generated using the other functional traits and phylogeny, or just phylogeny, compared to the distribution for non‐imputed values only. (b) shows the imputed maximum longevity values for species missing data, imputed with *missForest* and *Rphylopars*. *Rphylopars* contains more extreme values but has a more even distribution of values that closely match the distribution of non‐imputed values (a), whereas *missForest* has fewer extreme values but a less well‐matched distribution.
**Figure S3:** Pearson correlation values for each pair‐wise trait combination. Max longevity includes imputed values from *Rphylopars* imputation using traits and phylogeny.
**Figure S4:** Posterior predictive checks for each model, for (a–d) temperature as the predictor, and (e–h) time as the predictor. The models' predicted distributions (light blue) from 100 draws aligns well with the data distributions (dark blue) for all models, although relative redundancy (d, h) has a slightly underestimated peak.
**Figure S5:** Distribution of the standardised residuals for each model, for (a–d) temperature as the predictor, and (e–h) time as the predictor. All models agreed with the assumption of a normal distribution.
**Figure S6:** The distribution of the predicted values and standardised residuals for each model, for (a–d) temperature as the predictor, and (e–h) time as the predictor, suggesting equal variance (homoscedasticity) of the residuals.
**Figure S7:** Temporal autocorrelation of the standardised residuals for each model, for (a–d) temperature as the predictor, and (e–h) time as the predictor, suggesting little autocorrelation in the models.
**Figure S8:** Distribution of the contributions of the site random effect and fixed effects to temperature (a–d) and time (e–h) model responses to functional richness (a, e), divergence (b, f), evenness (c, g), and relative redundancy (d, h) differenced from the null model predictions. The site random effect did not significantly alter the model predictions.
**Figure S9:** Posterior predictions of functional divergence with scaled average winter temperature from 1000 random draws from the model, with the ribbons representing the 95% credible intervals. The part of Europe behind each facet grid square is the area the trend line represents, predicted from 25 longitude and latitude values on either side of the mid latitude and longitude.
**Figure S10:** Posterior predictions of functional evenness with scaled average winter temperature from 1000 random draws from the model, with the ribbons representing the 95% credible intervals. The part of Europe behind each facet grid square is the area the trend line represents, predicted from 25 longitude and latitude values on either side of the mid latitude and longitude.
**Figure S11:** Posterior predictions of functional evenness over time from 1000 random draws from the model, with the ribbons representing the 95% credible intervals. The part of Europe behind each facet grid square is the area the trend line represents, predicted from 25 longitude and latitude values on either side of the mid latitude and longitude.
**Figure S12:** Posterior predictions of relative redundancy over time from 1000 random draws from the model, with the ribbons representing the 95% credible intervals. The part of Europe behind each facet grid square is the area the trend line represents, predicted from 25 longitude and latitude values on either side of the mid latitude and longitude.
**Figure S13:** Posterior distributions from the winter temperature models, showing the effect of predictor variables and their interactions on functional richness, divergence, evenness, or relative redundancy. Unprotected areas are the reference level (intercept). Dots are the median and the whiskers are the 90% and 95% credible intervals, with faded colours representing distributions where the 95% credible interval overlaps zero (the grey dashed line), and bright colours representing distributions where the 95% credible interval does not overlap zero. Temp is the scaled average winter temperature, Lat is scaled latitude, Long is scaled longitude, Protected is non‐targeted protected areas and Ramsar/SPA are Ramsar sites or Special Protection Areas (Birds Directive).
**Figure S14:** Posterior distributions from the time models, showing the effect of predictor variables and their interactions on functional richness, divergence, evenness, or relative redundancy. Unprotected areas are the reference level (intercept). Dots are the median and the whiskers are the 90% and 95% credible intervals, with faded colours representing distributions where the 95% credible interval overlaps zero (the grey dashed line), and bright colours representing distributions where the 95% credible interval does not overlap zero. Year is the centred year, Lat is scaled latitude, Long is scaled longitude, Protected is non‐targeted protected areas and Ramsar/SPA are Ramsar sites or Special Protection Areas (Birds Directive).

## Data Availability

An exception to the data sharing policy was granted by the Editor‐in‐Chief. Waterbird count data must be requested from Wetlands International's International Waterbird Census online data portal (iwc.wetlands.org). The CHELSA V2.1 climate data is available at www.chelsa‐climate.org/datasets/chelsa_monthly. Protected Area data can be downloaded from the World Database on Protected Areas (www.protectedplanet.net/en/thematic‐areas/wdpa). The trait datasets used in our analysis can be downloaded from figshare repositories: Amniote (https://doi.org/10.6084/m9.figshare.c.3308127); EltonTraits (https://doi.org/10.6084/m9.figshare.c.3306933); and AVONET (http://figshare.com/s/b990722d72a26b5bfead, https://doi.org/10.1111/ele.13898). All code used in the analysis is available in the Zenodo repository https://doi.org/10.5281/zenodo.21807474.
