# Supplementary material for: Functional Diversity Responses to Temperature Vary Spatially and With Protection Status in Wintering Waterbird Assemblages
Source: Ecol Lett. 2026 Oct 20;29(10):e70467. doi: 10.1111/ele.70467 (PMC13682586; doi:10.1111/ele.70467)
Supplement: Supplementary file 1 [file ELE-29-0-s001.docx]

**Supplementary Material for:**

**Functional diversity responses to temperature vary spatially and with protection status in wintering waterbird assemblages**

Tanith B. Hackney^1^, Hannah S. Wauchope^2^, Pol Capdevila^3,4^, Christopher F. Clements^1^

^1^ School of Biological Sciences, University of Bristol, Bristol, UK

^2^ School of GeoSciences, The University of Edinburgh, Edinburgh, UK

^3^ Departament de Biologia Evolutiva, Ecologia i Ciències Ambientals, Facultat de Biologia, Universitat de Barcelona (UB), Barcelona, Spain

^4^ Institut de Recerca de la Biodiversitat (IRBio), Universitat de Barcelona (UB), Barcelona, Spain

Table of Contents

[Supplementary Methods	2](#_Toc228979562)

[**Data description**	2](#_Toc228979563)

[**Functional Traits Selection**	9](#_Toc228979564)

[**Trait Imputation**	12](#_Toc228979565)

[**Model Formulae**	15](#_Toc228979566)

[Model Diagnostics	18](#_Toc228979567)

[**Contribution of random versus fixed effects**	21](#_Toc228979568)

[Supplementary Results	23](#_Toc228979569)

[**Explained Variance**	27](#_Toc228979570)

[**Posterior Distribution Plots**	28](#_Toc228979571)

[**Model Coefficient Tables**	30](#_Toc228979572)

[References	38](#_Toc228979573)

## **Supplementary Methods**

### **Data description**

Our final dataset after cleaning included 213 waterbird species (Table S1). Our dataset included 168 resident species (classified as “breeding” or “resident”), with residents present at all 2792 of the sites, and 144 migrant species (classified as “passage” or “non-breeding”), with migrants present at 2758 of the sites, suggesting good representation of both migrants and residents across our dataset. There was representation across the entire study period (1980-2018), but the most common start year for a timeseries was 1980 (405 sites, Figure S1a). Overall, the latest start year for a timeseries was 2014, but the mean and median start year was 1997. All years were represented, with cumulative increase over time (Figure S1b). Our minimum time series length spanned 5 years, our maximum was 39 years, and the mean was 17 years (Figure S1c). Most sites had complete time series with no gaps in counts (1,388 timeseries), and the mean proportion of gaps for a site was 0.057 (Figure S1d).

**Table S1.** All 213 species in the final dataset used for the functional diversity calculations for the models. There were 250 species used for trait imputation, but we lost 37 species during the data cleaning steps after this, resulting in 213 species.

| **Family** | **Genus** | **Species** |
| --- | --- | --- |
| Anatidae | Aix | *Aix galericulata* |
| Anatidae | Aix | *Aix sponsa* |
| Anatidae | Alopochen | *Alopochen aegyptiaca* |
| Anatidae | Amazonetta | *Amazonetta brasiliensis* |
| Anatidae | Anas | *Anas acuta* |
| Anatidae | Anas | *Anas crecca* |
| Anatidae | Anas | *Anas platyrhynchos* |
| Anatidae | Anas | *Anas bahamensis* |
| Anatidae | Anas | *Anas poecilorhyncha* |
| Anatidae | Anas | *Anas superciliosa* |
| Anatidae | Anas | *Anas undulata* |
| Anatidae | Anas | *Anas capensis* |
| Anatidae | Anas | *Anas flavirostris* |
| Anatidae | Anas | *Anas rubripes* |
| Anatidae | Anas | *Anas castanea* |
| Anatidae | Anas | *Anas sparsa* |
| Anatidae | Anser | *Anser albifrons* |
| Anatidae | Anser | *Anser anser* |
| Anatidae | Anser | *Anser fabalis* |
| Anatidae | Anser | *Anser indicus* |
| Anatidae | Anser | *Anser brachyrhynchus* |
| Anatidae | Anser | *Anser cygnoid* |
| Anatidae | Anser | *Anser erythropus* |
| Anatidae | Anser | *Anser caerulescens* |
| Anatidae | Anser | *Anser rossii* |
| Anatidae | Anser | *Anser canagicus* |
| Anatidae | Aythya | *Aythya ferina* |
| Anatidae | Aythya | *Aythya fuligula* |
| Anatidae | Aythya | *Aythya marila* |
| Anatidae | Aythya | *Aythya nyroca* |
| Anatidae | Aythya | *Aythya collaris* |
| Anatidae | Aythya | *Aythya affinis* |
| Anatidae | Branta | *Branta leucopsis* |
| Anatidae | Branta | *Branta canadensis* |
| Anatidae | Branta | *Branta hutchinsii* |
| Anatidae | Branta | *Branta bernicla* |
| Anatidae | Branta | *Branta ruficollis* |
| Anatidae | Branta | *Branta sandvicensis* |
| Anatidae | Bucephala | *Bucephala clangula* |
| Anatidae | Bucephala | *Bucephala albeola* |
| Anatidae | Cairina | *Cairina moschata* |
| Anatidae | Callonetta | *Callonetta leucophrys* |
| Anatidae | Chenonetta | *Chenonetta jubata* |
| Anatidae | Chloephaga | *Chloephaga picta* |
| Anatidae | Chloephaga | *Chloephaga melanoptera* |
| Anatidae | Clangula | *Clangula hyemalis* |
| Anatidae | Coscoroba | *Coscoroba coscoroba* |
| Anatidae | Cygnus | *Cygnus olor* |
| Anatidae | Cygnus | *Cygnus atratus* |
| Anatidae | Cygnus | *Cygnus cygnus* |
| Anatidae | Cygnus | *Cygnus columbianus* |
| Anatidae | Cygnus | *Cygnus melancoryphus* |
| Anatidae | Dendrocygna | *Dendrocygna bicolor* |
| Anatidae | Dendrocygna | *Dendrocygna javanica* |
| Anatidae | Lophodytes | *Lophodytes cucullatus* |
| Anatidae | Mareca | *Mareca penelope* |
| Anatidae | Mareca | *Mareca strepera* |
| Anatidae | Mareca | *Mareca sibilatrix* |
| Anatidae | Mareca | *Mareca falcata* |
| Anatidae | Mareca | *Mareca americana* |
| Anatidae | Marmaronetta | *Marmaronetta angustirostris* |
| Anatidae | Melanitta | *Melanitta fusca* |
| Anatidae | Melanitta | *Melanitta nigra* |
| Anatidae | Melanitta | *Melanitta perspicillata* |
| Anatidae | Mergellus | *Mergellus albellus* |
| Anatidae | Mergus | *Mergus merganser* |
| Anatidae | Mergus | *Mergus serrator* |
| Anatidae | Netta | *Netta rufina* |
| Anatidae | Netta | *Netta peposaca* |
| Anatidae | Netta | *Netta erythrophthalma* |
| Anatidae | Oxyura | *Oxyura jamaicensis* |
| Anatidae | Oxyura | *Oxyura leucocephala* |
| Anatidae | Polysticta | *Polysticta stelleri* |
| Anatidae | Radjah | *Radjah radjah* |
| Anatidae | Somateria | *Somateria mollissima* |
| Anatidae | Somateria | *Somateria spectabilis* |
| Anatidae | Spatula | *Spatula clypeata* |
| Anatidae | Spatula | *Spatula querquedula* |
| Anatidae | Spatula | *Spatula rhynchotis* |
| Anatidae | Spatula | *Spatula versicolor* |
| Anatidae | Spatula | *Spatula cyanoptera* |
| Anatidae | Spatula | *Spatula discors* |
| Anatidae | Spatula | *Spatula hottentota* |
| Anatidae | Tadorna | *Tadorna tadorna* |
| Anatidae | Tadorna | *Tadorna ferruginea* |
| Anatidae | Tadorna | *Tadorna cana* |
| Anatidae | Tadorna | *Tadorna variegata* |
| Ardeidae | Ardea | *Ardea cinerea* |
| Ardeidae | Ardea | *Ardea alba* |
| Ardeidae | Ardea | *Ardea purpurea* |
| Ardeidae | Ardeola | *Ardeola ralloides* |
| Ardeidae | Botaurus | *Botaurus stellaris* |
| Ardeidae | Bubulcus | *Bubulcus ibis* |
| Ardeidae | Butorides | *Butorides striata* |
| Ardeidae | Egretta | *Egretta garzetta* |
| Ardeidae | Egretta | *Egretta gularis* |
| Ardeidae | Ixobrychus | *Ixobrychus minutus* |
| Ardeidae | Nycticorax | *Nycticorax nycticorax* |
| Burhinidae | Burhinus | *Burhinus oedicnemus* |
| Charadriidae | Charadrius | *Charadrius hiaticula* |
| Charadriidae | Charadrius | *Charadrius alexandrinus* |
| Charadriidae | Charadrius | *Charadrius dubius* |
| Charadriidae | Charadrius | *Charadrius leschenaultii* |
| Charadriidae | Eudromias | *Eudromias morinellus* |
| Charadriidae | Pluvialis | *Pluvialis apricaria* |
| Charadriidae | Pluvialis | *Pluvialis squatarola* |
| Charadriidae | Vanellus | *Vanellus vanellus* |
| Charadriidae | Vanellus | *Vanellus spinosus* |
| Charadriidae | Vanellus | *Vanellus gregarius* |
| Charadriidae | Vanellus | *Vanellus armatus* |
| Ciconiidae | Ciconia | *Ciconia nigra* |
| Ciconiidae | Ciconia | *Ciconia ciconia* |
| Gaviidae | Gavia | *Gavia adamsii* |
| Gaviidae | Gavia | *Gavia arctica* |
| Gaviidae | Gavia | *Gavia stellata* |
| Gaviidae | Gavia | *Gavia immer* |
| Gaviidae | Gavia | *Gavia pacifica* |
| Gruidae | Grus | *Grus grus* |
| Haematopodidae | Haematopus | *Haematopus ostralegus* |
| Laridae | Chlidonias | *Chlidonias niger* |
| Laridae | Chlidonias | *Chlidonias hybrida* |
| Laridae | Chlidonias | *Chlidonias leucopterus* |
| Laridae | Gelochelidon | *Gelochelidon nilotica* |
| Laridae | Hydrocoloeus | *Hydrocoloeus minutus* |
| Laridae | Hydroprogne | *Hydroprogne caspia* |
| Laridae | Larus | *Larus cachinnans* |
| Laridae | Larus | *Larus canus* |
| Laridae | Larus | *Larus michahellis* |
| Laridae | Larus | *Larus ridibundus* |
| Laridae | Larus | *Larus argentatus* |
| Laridae | Larus | *Larus fuscus* |
| Laridae | Larus | *Larus melanocephalus* |
| Laridae | Larus | *Larus ichthyaetus* |
| Laridae | Larus | *Larus marinus* |
| Laridae | Larus | *Larus pipixcan* |
| Laridae | Larus | *Larus hyperboreus* |
| Laridae | Larus | *Larus atricilla* |
| Laridae | Larus | *Larus genei* |
| Laridae | Larus | *Larus audouinii* |
| Laridae | Larus | *Larus delawarensis* |
| Laridae | Larus | *Larus philadelphia* |
| Laridae | Larus | *Larus glaucoides* |
| Laridae | Larus | *Larus armenicus* |
| Laridae | Rissa | *Rissa tridactyla* |
| Laridae | Sterna | *Sterna paradisaea* |
| Laridae | Sterna | *Sterna hirundo* |
| Laridae | Sterna | *Sterna forsteri* |
| Laridae | Sternula | *Sternula albifrons* |
| Laridae | Thalasseus | *Thalasseus sandvicensis* |
| Laridae | Xema | *Xema sabini* |
| Pelecanidae | Pelecanus | *Pelecanus crispus* |
| Pelecanidae | Pelecanus | *Pelecanus onocrotalus* |
| Phalacrocoracidae | Gulosus | *Gulosus aristotelis* |
| Phalacrocoracidae | Microcarbo | *Microcarbo pygmaeus* |
| Phalacrocoracidae | Phalacrocorax | *Phalacrocorax carbo* |
| Phoenicopteridae | Phoenicopterus | *Phoenicopterus chilensis* |
| Phoenicopteridae | Phoenicopterus | *Phoenicopterus roseus* |
| Phoenicopteridae | Phoeniconaias | *Phoeniconaias minor* |
| Phoenicopteridae | Phoenicopterus | *Phoenicopterus ruber* |
| Podicipedidae | Podiceps | *Podiceps cristatus* |
| Podicipedidae | Podiceps | *Podiceps nigricollis* |
| Podicipedidae | Podiceps | *Podiceps auritus* |
| Podicipedidae | Podiceps | *Podiceps grisegena* |
| Podicipedidae | Podilymbus | *Podilymbus podiceps* |
| Podicipedidae | Tachybaptus | *Tachybaptus ruficollis* |
| Rallidae | Fulica | *Fulica atra* |
| Rallidae | Fulica | *Fulica cristata* |
| Rallidae | Gallinula | *Gallinula chloropus* |
| Rallidae | Porphyrio | *Porphyrio porphyrio* |
| Rallidae | Porphyrio | *Porphyrio alleni* |
| Rallidae | Porzana | *Porzana porzana* |
| Rallidae | Rallus | *Rallus aquaticus* |
| Rallidae | Zapornia | *Zapornia parva* |
| Recurvirostridae | Himantopus | *Himantopus himantopus* |
| Recurvirostridae | Recurvirostra | *Recurvirostra avosetta* |
| Scolopacidae | Actitis | *Actitis hypoleucos* |
| Scolopacidae | Actitis | *Actitis macularius* |
| Scolopacidae | Arenaria | *Arenaria interpres* |
| Scolopacidae | Calidris | *Calidris alpina* |
| Scolopacidae | Calidris | *Calidris pugnax* |
| Scolopacidae | Calidris | *Calidris minuta* |
| Scolopacidae | Calidris | *Calidris temminckii* |
| Scolopacidae | Calidris | *Calidris alba* |
| Scolopacidae | Calidris | *Calidris canutus* |
| Scolopacidae | Calidris | *Calidris maritima* |
| Scolopacidae | Calidris | *Calidris ferruginea* |
| Scolopacidae | Calidris | *Calidris mauri* |
| Scolopacidae | Gallinago | *Gallinago gallinago* |
| Scolopacidae | Gallinago | *Gallinago media* |
| Scolopacidae | Limnodromus | *Limnodromus scolopaceus* |
| Scolopacidae | Limosa | *Limosa lapponica* |
| Scolopacidae | Limosa | *Limosa limosa* |
| Scolopacidae | Lymnocryptes | *Lymnocryptes minimus* |
| Scolopacidae | Numenius | *Numenius arquata* |
| Scolopacidae | Numenius | *Numenius phaeopus* |
| Scolopacidae | Phalaropus | *Phalaropus fulicarius* |
| Scolopacidae | Phalaropus | *Phalaropus lobatus* |
| Scolopacidae | Scolopax | *Scolopax rusticola* |
| Scolopacidae | Steganopus | *Steganopus tricolor* |
| Scolopacidae | Tringa | *Tringa ochropus* |
| Scolopacidae | Tringa | *Tringa totanus* |
| Scolopacidae | Tringa | *Tringa stagnatilis* |
| Scolopacidae | Tringa | *Tringa erythropus* |
| Scolopacidae | Tringa | *Tringa glareola* |
| Scolopacidae | Tringa | *Tringa nebularia* |
| Scolopacidae | Tringa | *Tringa melanoleuca* |
| Scolopacidae | Tringa | *Tringa flavipes* |
| Scolopacidae | Xenus | *Xenus cinereus* |
| Threskiornithidae | Geronticus | *Geronticus eremita* |
| Threskiornithidae | Platalea | *Platalea leucorodia* |
| Threskiornithidae | Platalea | *Platalea alba* |
| Threskiornithidae | Plegadis | *Plegadis falcinellus* |
| Threskiornithidae | Threskiornis | *Threskiornis aethiopicus* |

**Figure S1.** (a) The frequency of year in which the time series starts. The highest start year was 1980 but there is good representation across all years with peaks in 1995 and 2011. (b) Number of sites in each year. (c) The length of time series. Most time series lengths are well represented in the dataset. (d) Proportion of missing years (gaps) in time series. Most time series had no gaps.

### **Functional Traits Selection**

#### Hand-wing Index

Hand-wing index (HWI) is calculated from the wing length and secondary feather length. It is correlated with the wing aspect ratio, so HWI can be used as a proxy for a bird’s flight and dispersal ability (Claramunt et al. 2012; Lockwood et al. 1998). This is important for ecological and evolutionary processes at multiple scales, from seed dispersal (Viana et al. 2016) to speciation and extinction patterns (Kisel and Barraclough 2010). Higher HWI is also associated with lower territoriality, higher seasonal and climatic variability, and migration in birds (Sheard et al. 2020), suggesting HWI is an indicator of flight efficiency and the ability to respond to environmental change (Bregman et al. 2014; Habel et al. 2019; Travis et al. 2013).

#### Body Mass

Body mass is strongly associated with ecological processes such as energy flow between trophic levels, food web structure, and processes such as seed dispersal, predation, or pollination, along with life history and metabolic rate (Brown et al. 1978; Cardillo et al. 2005; Porter and Kearney 2009). However, body size can also influence how a species responds to environmental change because it affects dispersal ability, space use and home range size, and vulnerability to certain disturbances, such as rising temperature or hunting pressure (Cardillo et al. 2005; Gardner et al. 2011). It can also be an important predictor of evolutionary rate, and has been linked to speed and extent of bird species’ phenotypic responses to climate change (Zimova et al. 2023).

#### Bill Morphology (length, width, and depth)

Bill morphology, including bill length, depth, and width, has been linked to foraging behaviour, strategy, and diet (Fitzpatrick 1985; Yu et al. 2023). For example, bill size has been associated with prey size (Schoener 1971), and as a result, foraging time (Zwarts et al. 2023). Bill morphology also shows strong correlations with trophic level and foraging niche (Pigot et al. 2020), and is important for thermoregulation (Probst et al. 2022). Bill shape and size can also influence responses to changes in food supply and climate (Probst et al. 2022; Zhang et al. 2019).

#### Tarsus Length

Similar to bill morphological traits, tarsus length influences foraging behaviour and diet (Fitzpatrick 1985; Miles and Ricklefs 1984), and has been linked to dietary guild, foraging niche, and feeding strategy (Aarif et al. 2024; Pigot et al. 2016). Tarsus length is also associated with habitat type/breadth and primary lifestyle (Tobias et al. 2022). For example, in wading birds, species with longer legs may be able to exploit more food sources than those with shorter legs (Linhart et al. 2022; Norazlimi and Ramli 2015; Ntiamoa-Baidu et al. 1998).

#### Clutch Size

Clutch size is related to numerous aspects of life history, such as development mode and nest type, as well as environmental factors such as seasonality and resource availability (Jetz et al. 2012). Clutch size can also affect ecosystem processes such as trophic interactions and resource consumption (Newbold et al. 2013). Responses to environmental change are also correlated with life strategy, with fecundity affecting a population’s ability to recover after disturbance (Albaladejo-Roble et al. 2023), with greater diversity in strategies improving a community’s ability to respond to change (Larsen et al. 2005).

*Diet Breadth*

Diet is important as it influences species and trophic interactions, metabolic rate, and determines ecological processes such as predation or herbivory (Brown et al. 2004; Burin et al. 2016; Duffy 2002). A species’ diet breadth also influences their response to environmental change. For example, it can affect whether and to what extent a species can make use of new resources or switch food sources with ecosystem change (Ducatez et al. 2015; Korpimäki et al. 2020; Newbold et al. 2013). To calculate diet breadth, we summed the diet categories containing values from EltonTraits (Wilman et al. 2014), so for a species that can eat three out of the 10 food types, that species has a diet breadth of three.

#### Maximum Longevity

Lifespan is linked to other life history traits (Promislow and Harvey 1990), which are important in determining responses to environmental change (Li et al. 2021) and ecosystem function (Hordley et al. 2021). Lifespan is also linked to migratory strategy (Soriano-Redondo et al. 2020). Longevity is correlated with other avian traits such as brain size (Minias and Podlaszczuk 2017), which has been demonstrated to be important in determining vulnerability to climate change (Keum et al. 2025).

### **Trait Imputation**

Numerous imputation methods exist for imputing missing trait values. We chose to impute traits using two R packages, *missForest* version 1.5 (Stekhoven 2022; Stekhoven and Bühlmann 2012) and *Rphylopars* (Goolsby et al. 2024) because both have shown promising results for imputation accuracy (Johnson et al. 2021; Penone et al. 2014). Including phylogeny can improve imputation accuracy so we generated a set of 1000 trees from BirdTree.org (Jetz et al. 2012) for the 250 species in our initial dataset, using the “Hackett All Species: a set of 10000 trees with 9993 OTUs each” option (Hackett et al. 2008). Based on visual inspection of the imputed and non-imputed values, we chose *Rphylopars* based on both traits and phylogeny because it most closely followed the distribution of the non-imputed values (Figure S2). *Rphylopars* has also been found to outperform other imputation methods for trait data (Johnson et al. 2021).

Correlation between traits does not have much effect on functional diversity metrics except at high correlation values (Lefcheck et al. 2015; Mammola and Cardoso 2020; Ohlert et al. 2024), with one cut-off for estimate KDE suggested at |r|>0.8 (Mammola and Cardoso 2020). Only two of our chosen traits were highly correlated (|r|=0.83) and this was beak width and beak depth. We removed beak depth as this was slightly more correlated with the other traits, leaving eight traits (Figure S3).

**Figure S2.** Comparison of maximum longevity distributions for each imputation method, *Rphylopars* and *missForest*, with and without traits and phylogeny. (a) shows the frequency distributions for all maximum longevity values for *missForest* and *Rphylopars*, generated using the other functional traits and phylogeny, or just phylogeny, compared to the distribution for non-imputed values only. (b) shows the imputed maximum longevity values for species missing data, imputed with *missForest* and *Rphylopars*. *Rphylopars* contains more extreme values but has a more even distribution of values that closely match the distribution of non-imputed values (a), whereas *missForest* has fewer extreme values but a less well-matched distribution.

**Figure S3.** Pearson correlation values for each pair-wise trait combination. Max longevity includes imputed values from *Rphylopars* imputation using traits and phylogeny.

### **Model Formulae**

We had four model structures. Model 1 (effect of temperature on functional richness) has the likelihood:

$$y_{i}\sim\text{Normal}(\mu_{i},\sigma)$$

And model:

$$\mu_{i}\text{ }=\text{ }\alpha+\mathbf{x}_{i}^{\top}\boldsymbol{\beta}+a_{s\left[ i \right]}^{\left( \text{site} \right)}+a_{t\left[ i \right]}^{\left( \text{year} \right)}+\eta_{s\left[ i \right],t\left[ i \right]}$$

$$\eta_{s,t}=\varphi\text{ }\eta_{s,t-1}+\epsilon_{s,t}$$

With priors:

$$\beta_{k}\sim\text{Normal}\left( 0,1 \right)$$

$$a_{s}^{\left( \text{site} \right)}\sim\text{Normal}\left( 0,\sigma_{\text{site}} \right)$$

$$a_{t}^{\left( \text{year} \right)}\sim\text{Normal}\left( 0,\sigma_{\text{year}} \right)$$

$$\sigma_{\text{site}}\sim\text{Exponential}\left( 1 \right)$$

$$\sigma_{\text{year}}\sim\text{Exponential}\left( 1 \right)$$

$$\varphi\sim\text{Normal}(0,0.25)$$

$$\epsilon_{s,t}\sim\text{Normal}\left( 0,\sigma_{\eta} \right)$$

Where $\mu_{i}$ is the mean and $\sigma$ is the standard deviation. $\mathbf{x}_{i}$ represents the fixed effects (Average winter temperature, Latitude, Longitude$\text{,}$ and ProtectionStatus) and all interactions, with $\boldsymbol{\beta}$ the coefficient for the fixed effects. $a_{s}^{\left( \text{site} \right)}$is the random intercept for site $s$, $a_{t}^{\left( \text{year} \right)}$is the random intercept for year $t$, and $\eta_{i}$is the AR(1) time-series residual for observation $i$within site $s$. $\eta_{s,t}$ is the residual associated with site $s$ and year $t$.

Models 2, 3, and 4 (effect of temperature on functional divergence, evenness, or redundancy) are:

$$z_{i}\sim\text{OrderedBeta}\left( \mu_{i},\phi,\boldsymbol{\kappa} \right)$$

$$\text{logit}\left( \mu_{i} \right)\text{ }=\alpha+\mathbf{x}_{i}^{\top}\boldsymbol{\beta}+a_{s\left[ i \right]}^{\left( \text{site} \right)}+a_{t\left[ i \right]}^{\left( \text{year} \right)}+\eta_{i}$$

$$\eta_{s,t}=\phi_{\text{AR}}\eta_{s,t-1}+\epsilon_{s,t}$$

Priors:

$$a_{s}^{\left( \text{site} \right)}\sim\text{Normal}\left( 0,\sigma_{\text{site}} \right)$$

$$a_{t}^{\left( \text{year} \right)}\sim\text{Normal}\left( 0,\sigma_{\text{year}} \right)$$

$$\sigma_{\text{site}}\sim\text{Exponential}\left( 1 \right)$$

$$\sigma_{\text{year}}\sim\text{Exponential}(1)$$

$$\epsilon_{s,t}\sim\text{Normal}\left( 0,\sigma_{\eta} \right)$$

$$\phi_{\text{AR}}\sim\text{Normal}\left( 0,0.25 \right)$$

$$\phi\sim\text{Exponential}(1)$$

This is similar to Model 1 but $\mathbf{x}_{i}$ is modeled with an ordered beta distribution with logit link for the mean and an Exponential(1) prior on the dispersion parameter $\phi$. $\mu_{i}\in(0,1)$ is the mean on the Beta scale, $\phi>0$is the dispersion parameter, and $\kappa$ are cut points that determine the probabilities of exact 0, continuous (0,1), and exact 1. We used the default Dirichlet prior (Dirichlet(1,1,1) in *ordbetareg* for the cut points $\boldsymbol{\kappa}=\left( \kappa_{1},\kappa_{2} \right)$.

Model 5 (effect of time on functional richness) is:

$$y_{i}\sim\text{Normal}(\mu_{i},\sigma)$$

$$\mu_{i}=\text{ }\alpha+\mathbf{z}_{i}^{\top}\boldsymbol{\beta}+a_{s\left[ i \right]}^{\left( \text{site} \right)}+b_{s\left[ i \right]}^{\left( \text{site} \right)}\text{ }\text{CY}_{i}+\eta_{s\left[ i \right],t\left[ i \right]}$$

$$\eta_{s,t}=\varphi\text{ }\eta_{s,t-1}+\epsilon_{s,t}$$

Priors:

$$\beta_{k}\sim\text{Normal}\left( 0,1 \right)$$

$$\left( \begin{aligned} a_{s}^{\left( \text{site} \right)} \\ b_{s}^{\left( \text{site} \right)} \end{aligned} \right)\sim\text{MVNormal}\left( \left( \begin{aligned} 0 \\ 0 \end{aligned} \right) , \boldsymbol{\Sigma}_{\text{site}} \right)$$

$$\boldsymbol{\Sigma}_{\text{site}}=\left( \begin{matrix} \sigma_{a,\text{site}}^{2} & \rho_{\text{site}}\sigma_{a,\text{site}}\sigma_{b,\text{site}} \\ \rho_{\text{site}}\sigma_{a,\text{site}}\sigma_{b,\text{site}} & \sigma_{b,\text{site}}^{2} \end{matrix} \right)$$

$$\epsilon_{s,t}\sim\text{Normal}\left( 0,\sigma_{\eta} \right)$$

$$\phi\sim\text{Normal}(0,0.25)$$

$$\sigma_{b,\text{site}}\sim\text{Exponential}\left( 1 \right)$$

$$\varphi\sim\text{Normal}(0,0.25)$$

This is similar to Model 1 but $\text{CY}_{i}$ is the fixed effect centred year (CentreYear), and $\mathbf{z}_{i}$ contains the other fixed effects (Latitude, Longitude, ProtectionStatus) and their interactions. $a_{s}^{\left( \text{site} \right)}$is still the site-specific intercept, but $b_{s}^{\left( \text{site} \right)}$is the site-specific slope of CY.

Models 6, 7, and 8 are similar to Models 2, 3, and 4 but this time looking at effect of time on functional divergence, evenness, or redundancy so we had a random intercept and random slope for CentreYear at site $s$:

$$z_{i}\sim\text{OrderedBeta}\left( \mu_{i},\phi,\boldsymbol{\kappa} \right)$$

$$\text{logit}(\mu_{i})\text{ }=\text{ }\alpha+\mathbf{z}_{i}^{\top}\boldsymbol{\beta}+\left( a_{s[i]}^{\left( \text{site} \right)} + b_{s[i]}^{\left( \text{site} \right)}\text{ }\text{CY}_{i} \right)+\eta_{i}$$

$$\eta_{s,t}=\phi_{\text{AR}}\eta_{s,t-1}+\epsilon_{s,t}$$

Priors:

$$\left( \begin{aligned} a_{s}^{\left( \text{site} \right)} \\ b_{s}^{\left( \text{site} \right)} \end{aligned} \right)\sim\text{MVNormal}\left( \left( \begin{aligned} 0 \\ 0 \end{aligned} \right) , \boldsymbol{\Sigma}_{\text{site}} \right)$$

$$\boldsymbol{\Sigma}_{\text{site}}=\left( \begin{matrix} \sigma_{a,\text{site}}^{2} & \rho_{\text{site}}\sigma_{a,\text{site}}\sigma_{b,\text{site}} \\ \rho_{\text{site}}\sigma_{a,\text{site}}\sigma_{b,\text{site}} & \sigma_{b,\text{site}}^{2} \end{matrix} \right)$$

$$\sigma_{a,\text{site}}\sim\text{Exponential}\left( 1 \right)$$

$$\sigma_{b,\text{site}}\sim\text{Exponential}\left( 1 \right)$$

$$\rho_{\text{site}}\sim\text{LKJ}\left( 2 \right)$$

$$\epsilon_{s,t}\sim\text{Normal}\left( 0,\sigma_{\eta} \right)$$

$$\phi_{\text{AR}}\sim\text{Normal}\left( 0,0.25 \right)$$

$$\phi\sim\text{Exponential}\left( 1 \right)$$

## **Model Diagnostics**

All models had variance inflation factors below three (Tables S2-S3), indicating low multicollinearity (Zuur et al. 2010).

**Table S2.** Variance inflation factors (VIF) with 95% confidence for each of the models with temperature as a predictor. All have low correlation.

| Term | FRic  VIF (95% CI) | FDiv  VIF (95% CI) | FEve  VIF (95% CI) | RRed  VIF (95% CI) |
| --- | --- | --- | --- | --- |
| Seasonal_tas_scaled | 1.56 (1.54, 1.58) | 1.98 (1.96, 2.01) | 1.88 (1.85, 1.90) | 2.19 (2.16, 2.22) |
| Latitude_scaled | 1.39 (1.38, 1.41) | 1.92 (1.90, 1.94) | 1.88 (1.85, 1.90) | 1.94 (1.92, 1.97) |
| Longitude_scaled | 1.44 (1.42, 1.46) | 2.14 (2.11, 2.17) | 1.93 (1.90, 1.95) | 2.24 (2.21, 2.27) |
| ProtectionStatus | 1.17 (1.16, 1.18) | 2.33 (2.30, 2.36) | 2.07 (2.05, 2.10) | 2.38 (2.35, 2.41) |

**Table S3.** Variance inflation factors (VIF) with 95% confidence for each of the models with time as a predictor. All have low correlation.

| Term | FRic  VIF (95% CI) | FDiv  VIF (95% CI) | FEve  VIF (95% CI) | RRed  VIF (95% CI) |
| --- | --- | --- | --- | --- |
| CentreYear | 1.40 (1.39, 1.42) | 1.37 (1.35, 1.38) | 1.37 (1.35, 1.38) | 1.34 (1.33, 1.35) |
| Latitude_scaled | 1.18 (1.17 1.19) | 1.44 (1.43, 1.46) | 1.46 (1.45, 1.48) | 1.66 (1.64, 1.68) |
| Longitude_scaled | 1.24 (1.23, 1.25) | 1.51 (1.49, 1.52) | 1.55 (1.53, 1.56) | 1.95 (1.93, 1.97) |
| ProtectionStatus | 1.13 (1.12, 1.14) | 1.48 (1.46, 1.49) | 1.54 (1.52, 1.55) | 2.54 (2.50, 2.57) |

**Figure S4.** Posterior predictive checks for each model, for (a-d) temperature as the predictor, and (e-h) time as the predictor. The models’ predicted distributions (light blue) from 100 draws aligns well with the data distributions (dark blue) for all models, although relative redundancy (d, h) has a slightly underestimated peak.

**Figure S5.** Distribution of the standardised residuals for each model, for (a-d) temperature as the predictor, and (e-h) time as the predictor. All models agreed with the assumption of a normal distribution.

**Figure S6.** The distribution of the predicted values and standardised residuals for each model, for (a-d) temperature as the predictor, and (e-h) time as the predictor, suggesting equal variance (homoscedasticity) of the residuals.

**Figure S7.** Temporal autocorrelation of the standardised residuals for each model, for (a-d) temperature as the predictor, and (e-h) time as the predictor, suggesting little autocorrelation in the models.

### **Contribution of random versus fixed effects**

We compared the variation of functional diversity or redundancy responses predicted using only the site random effect to the variation predicted by the fixed effects (Fig S8; Capdevila et al. 2026). We differenced the posterior predictions made with a random effect model (generated from the null model including site random effect) or fixed effect model (where random effects are set to “0”), from the null model (by setting all random and fixed effects to “0” or reference levels), allowing us to generate a distribution of contributions. All of these overlapped with zero, so the site random effect is not contributing significantly to the direction of the responses for either the temperature models (Fig. S8 a-d) or time models (Fig. S8 e-h).

**Figure S8.** Distribution of the contributions of the site random effect and fixed effects to temperature (a-d) and time (e-h) model responses to functional richness (a, e), divergence (b, f), evenness (c, g), and relative redundancy (d, h) differenced from the null model predictions. The site random effect did not significantly alter the model predictions.

## **Supplementary Results**

**Figure S9.** Posterior predictions of functional divergence with scaled average winter temperature from 1000 random draws from the model, with the ribbons representing the 95% credible intervals. The part of Europe behind each facet grid square is the area the trend line represents, predicted from 25 longitude and latitude values on either side of the mid latitude and longitude.

**Figure S10.** Posterior predictions of functional evenness with scaled average winter temperature from 1000 random draws from the model, with the ribbons representing the 95% credible intervals. The part of Europe behind each facet grid square is the area the trend line represents, predicted from 25 longitude and latitude values on either side of the mid latitude and longitude.

**Figure S11.** Posterior predictions of functional evenness over time from 1000 random draws from the model, with the ribbons representing the 95% credible intervals. The part of Europe behind each facet grid square is the area the trend line represents, predicted from 25 longitude and latitude values on either side of the mid latitude and longitude.

**Figure S12.** Posterior predictions of relative redundancy over time from 1000 random draws from the model, with the ribbons representing the 95% credible intervals. The part of Europe behind each facet grid square is the area the trend line represents, predicted from 25 longitude and latitude values on either side of the mid latitude and longitude.

### **Explained Variance**

We used an R^2^ suitable for Bayesian regression models (Gelman et al. 2019) to summarise the variance explained by each of our models and assess model fit. Bayesian R^2^ ranged from 55% to 75% in the models with temperature as a predictor, and 58% to 77% in the models with time as a predictor (Table S4). Overall, the models explained a moderate amount of variance, but the relative redundancy model explained the most amount of variance in both the temperature and time models.

**Table S4.** Bayesian R^2^ with 95% credible intervals for each of the models with temperature or time as a predictor, and functional richness, divergence, evenness, or redundancy as the response variable.

| Predictor | FRic  R^2^ (95% CI) | FDiv  R^2^ (95% CI) | FEve  R^2^ (95% CI) | RRed  R^2^ (95% CI) |
| --- | --- | --- | --- | --- |
| Temperature | 0.55 (0.55, 0.56) | 0.62 (0.59, 0.64) | 0.59 (0.57, 0.61) | 0.75 (0.74, 0.76) |
| Time | 0.58 (0.58, 0.58) | 0.61 (0.58, 0.63) | 0.58 (0.56, 0.60) | 0.77 (0.76, 0.77) |

### **Posterior Distribution Plots**

**Figure S13.** Posterior distributions from the winter temperature models, showing the effect of predictor variables and their interactions on functional richness, divergence, evenness, or relative redundancy. Unprotected areas are the reference level (intercept). Dots are the median and the whiskers are the 90 and 95% credible intervals, with faded colours representing distributions where the 95% credible interval overlaps zero (the grey dashed line), and bright colours representing distributions where the 95% credible interval does not overlap zero. Temp is the scaled average winter temperature, Lat is scaled latitude, Long is scaled longitude, Protected is non-targeted protected areas and Ramsar/SPA are Ramsar sites or Special Protection Areas (Birds Directive).

**Figure S14.** Posterior distributions from the time models, showing the effect of predictor variables and their interactions on functional richness, divergence, evenness, or relative redundancy. Unprotected areas are the reference level (intercept). Dots are the median and the whiskers are the 90 and 95% credible intervals, with faded colours representing distributions where the 95% credible interval overlaps zero (the grey dashed line), and bright colours representing distributions where the 95% credible interval does not overlap zero. Year is the centred year, Lat is scaled latitude, Long is scaled longitude, Protected is non-targeted protected areas and Ramsar/SPA are Ramsar sites or Special Protection Areas (Birds Directive).

### **Model Coefficient Tables**

**Table S5.** The coefficients from the functional richness and temperature model, including the median and lower and upper 95% credible intervals of the posterior distribution. Rhat close to one indicates convergence, and ESS is the effective sample size.

| Parameter | Median | Lower CI (95%) | Upper CI (95%) | Rhat | ESS |
| --- | --- | --- | --- | --- | --- |
| Intercept | 109.464 | 104.383 | 114.366 | 1.01 | 433 |
| Seasonal_tas_scaled | 1.763 | 1.041 | 2.481 | 1.00 | 5539 |
| Latitude_scaled | 0.222 | -0.699 | 1.154 | 1.00 | 2319 |
| Longitude_scaled | -1.144 | -2.023 | -0.265 | 1.00 | 2788 |
| ProtectionStatusProtected | 0.448 | -1.110 | 2.012 | 1.00 | 4433 |
| ProtectionStatusRamsarSPA | -1.000 | -2.631 | 0.614 | 1.00 | 4680 |
| Seasonal_tas_scaled:Latitude_scaled | 0.228 | -0.296 | 0.747 | 1.00 | 3819 |
| Seasonal_tas_scaled:Longitude_scaled | -0.614 | -1.145 | -0.095 | 1.00 | 6010 |
| Latitude_scaled:Longitude_scaled | -1.905 | -2.666 | -1.118 | 1.00 | 2509 |
| Seasonal_tas_scaled:ProtectionStatusProtected | 1.144 | -0.010 | 2.313 | 1.00 | 8795 |
| Seasonal_tas_scaled:ProtectionStatusRamsarSPA | 1.947 | 0.768 | 3.137 | 1.00 | 9519 |
| Latitude_scaled:ProtectionStatusProtected | -0.420 | -2.185 | 1.266 | 1.00 | 6710 |
| Latitude_scaled:ProtectionStatusRamsarSPA | -0.809 | -2.423 | 0.874 | 1.00 | 5542 |
| Longitude_scaled:ProtectionStatusProtected | 0.160 | -1.388 | 1.683 | 1.00 | 4206 |
| Longitude_scaled:ProtectionStatusRamsarSPA | -0.114 | -1.609 | 1.347 | 1.00 | 4996 |
| Seasonal_tas_scaled:Latitude_scaled:Longitude_scaled | 0.639 | 0.267 | 0.999 | 1.00 | 3940 |
| Seasonal_tas_scaled:Latitude_scaled:ProtectionStatusProtected | 0.544 | -0.811 | 1.842 | 1.00 | 7196 |
| Seasonal_tas_scaled:Latitude_scaled:ProtectionStatusRamsarSPA | 1.095 | 0.049 | 2.142 | 1.00 | 5668 |
| Seasonal_tas_scaled:Longitude_scaled:ProtectionStatusProtected | 1.364 | 0.257 | 2.480 | 1.00 | 6915 |
| Seasonal_tas_scaled:Longitude_scaled:ProtectionStatusRamsarSPA | 1.027 | -0.140 | 2.133 | 1.00 | 7588 |
| Latitude_scaled:Longitude_scaled:ProtectionStatusProtected | 1.103 | -0.459 | 2.647 | 1.00 | 5263 |
| Latitude_scaled:Longitude_scaled:ProtectionStatusRamsarSPA | -0.444 | -1.892 | 1.023 | 1.00 | 4444 |
| Seasonal_tas_scaled:Latitude_scaled:Longitude_scaled:ProtectionStatusProtected | 0.165 | -0.933 | 1.205 | 1.00 | 6183 |
| Seasonal_tas_scaled:Latitude_scaled:Longitude_scaled:ProtectionStatusRamsarSPA | 0.140 | -0.667 | 0.905 | 1.00 | 5121 |

**Table S6.** The coefficients from the functional divergence and temperature model, including the median and lower and upper 95% credible intervals of the posterior distribution. Rhat close to one indicates convergence, and ESS is the effective sample size.

| Parameter | Median | Lower CI (95%) | Upper CI (95%) | Rhat | ESS |
| --- | --- | --- | --- | --- | --- |
| Intercept | 0.765 | 0.738 | 0.794 | 1.00 | 562 |
| Seasonal_tas_scaled | 0.011 | -0.003 | 0.024 | 1.00 | 2194 |
| Latitude_scaled | -0.046 | -0.063 | -0.028 | 1.01 | 821 |
| Longitude_scaled | -0.026 | -0.042 | -0.009 | 1.00 | 917 |
| ProtectionStatusProtected | -0.016 | -0.060 | 0.030 | 1.00 | 899 |
| ProtectionStatusRamsarSPA | -0.081 | -0.128 | -0.037 | 1.00 | 966 |
| Seasonal_tas_scaled:Latitude_scaled | 0.027 | 0.017 | 0.036 | 1.00 | 1659 |
| Seasonal_tas_scaled:Longitude_scaled | -0.002 | -0.011 | 0.008 | 1.00 | 2108 |
| Latitude_scaled:Longitude_scaled | 0.009 | -0.005 | 0.023 | 1.00 | 1271 |
| Seasonal_tas_scaled:ProtectionStatusProtected | -0.003 | -0.028 | 0.022 | 1.00 | 3606 |
| Seasonal_tas_scaled:ProtectionStatusRamsarSPA | -0.024 | -0.052 | 0.004 | 1.00 | 4091 |
| Latitude_scaled:ProtectionStatusProtected | 0.007 | -0.056 | 0.068 | 1.00 | 1273 |
| Latitude_scaled:ProtectionStatusRamsarSPA | -0.013 | -0.063 | 0.036 | 1.00 | 1170 |
| Longitude_scaled:ProtectionStatusProtected | -0.005 | -0.049 | 0.039 | 1.00 | 1161 |
| Longitude_scaled:ProtectionStatusRamsarSPA | 0.007 | -0.032 | 0.048 | 1.00 | 996 |
| Seasonal_tas_scaled:Latitude_scaled:Longitude_scaled | -0.003 | -0.010 | 0.003 | 1.00 | 1833 |
| Seasonal_tas_scaled:Latitude_scaled:ProtectionStatusProtected | -0.009 | -0.042 | 0.023 | 1.00 | 2143 |
| Seasonal_tas_scaled:Latitude_scaled:ProtectionStatusRamsarSPA | 0.011 | -0.013 | 0.035 | 1.00 | 2151 |
| Seasonal_tas_scaled:Longitude_scaled:ProtectionStatusProtected | -0.015 | -0.040 | 0.010 | 1.00 | 2500 |
| Seasonal_tas_scaled:Longitude_scaled:ProtectionStatusRamsarSPA | -0.007 | -0.032 | 0.020 | 1.00 | 2510 |
| Latitude_scaled:Longitude_scaled:ProtectionStatusProtected | 0.034 | -0.012 | 0.081 | 1.00 | 1278 |
| Latitude_scaled:Longitude_scaled:ProtectionStatusRamsarSPA | 0.008 | -0.029 | 0.044 | 1.00 | 1286 |
| Seasonal_tas_scaled:Latitude_scaled:Longitude_scaled:ProtectionStatusProtected | -0.012 | -0.035 | 0.011 | 1.00 | 2195 |
| Seasonal_tas_scaled:Latitude_scaled:Longitude_scaled:ProtectionStatusRamsarSPA | -0.015 | -0.032 | 0.002 | 1.00 | 1811 |

**Table S7.** The coefficients from the functional evenness and temperature model, including the median and lower and upper 95% credible intervals of the posterior distribution. Rhat close to one indicates convergence, and ESS is the effective sample size.

| Parameter | Median | Lower CI (95%) | Upper CI (95%) | Rhat | ESS |
| --- | --- | --- | --- | --- | --- |
| Intercept | -0.723 | -0.736 | -0.710 | 1.00 | 896 |
| Seasonal_tas_scaled | 0.011 | 0.004 | 0.018 | 1.00 | 2958 |
| Latitude_scaled | 0.029 | 0.019 | 0.039 | 1.00 | 977 |
| Longitude_scaled | -0.052 | -0.061 | -0.043 | 1.00 | 1345 |
| ProtectionStatusProtected | 0.012 | -0.012 | 0.036 | 1.00 | 1373 |
| ProtectionStatusRamsarSPA | -0.040 | -0.063 | -0.015 | 1.00 | 1110 |
| Seasonal_tas_scaled:Latitude_scaled | -0.008 | -0.013 | -0.003 | 1.00 | 2429 |
| Seasonal_tas_scaled:Longitude_scaled | 0.007 | 0.002 | 0.012 | 1.00 | 4111 |
| Latitude_scaled:Longitude_scaled | -0.011 | -0.019 | -0.003 | 1.00 | 1427 |
| Seasonal_tas_scaled:ProtectionStatusProtected | -0.001 | -0.014 | 0.013 | 1.00 | 4836 |
| Seasonal_tas_scaled:ProtectionStatusRamsarSPA | -0.014 | -0.029 | 0.001 | 1.00 | 5590 |
| Latitude_scaled:ProtectionStatusProtected | 0.003 | -0.033 | 0.038 | 1.00 | 1370 |
| Latitude_scaled:ProtectionStatusRamsarSPA | -0.027 | -0.055 | 0.000 | 1.00 | 1533 |
| Longitude_scaled:ProtectionStatusProtected | -0.008 | -0.031 | 0.015 | 1.00 | 1758 |
| Longitude_scaled:ProtectionStatusRamsarSPA | 0.007 | -0.015 | 0.028 | 1.00 | 1367 |
| Seasonal_tas_scaled:Latitude_scaled:Longitude_scaled | -0.003 | -0.006 | 0.001 | 1.00 | 2673 |
| Seasonal_tas_scaled:Latitude_scaled:ProtectionStatusProtected | -0.006 | -0.024 | 0.012 | 1.00 | 2735 |
| Seasonal_tas_scaled:Latitude_scaled:ProtectionStatusRamsarSPA | -0.001 | -0.014 | 0.012 | 1.00 | 2514 |
| Seasonal_tas_scaled:Longitude_scaled:ProtectionStatusProtected | -0.006 | -0.019 | 0.007 | 1.00 | 4193 |
| Seasonal_tas_scaled:Longitude_scaled:ProtectionStatusRamsarSPA | 0.000 | -0.014 | 0.014 | 1.00 | 3802 |
| Latitude_scaled:Longitude_scaled:ProtectionStatusProtected | -0.025 | -0.050 | 0.000 | 1.00 | 1688 |
| Latitude_scaled:Longitude_scaled:ProtectionStatusRamsarSPA | 0.005 | -0.015 | 0.025 | 1.00 | 1752 |
| Seasonal_tas_scaled:Latitude_scaled:Longitude_scaled:ProtectionStatusProtected | 0.004 | -0.009 | 0.016 | 1.00 | 2938 |
| Seasonal_tas_scaled:Latitude_scaled:Longitude_scaled:ProtectionStatusRamsarSPA | 0.001 | -0.008 | 0.010 | 1.00 | 2672 |

**Table S8.** The coefficients from the relative redundancy and temperature model, including the median and lower and upper 95% credible intervals of the posterior distribution. Rhat close to one indicates convergence, and ESS is the effective sample size.

| Parameter | Median | Lower CI (95%) | Upper CI (95%) | Rhat | ESS |
| --- | --- | --- | --- | --- | --- |
| Intercept | -1.702 | -1.771 | -1.628 | 1.01 | 526 |
| Seasonal_tas_scaled | -0.032 | -0.056 | -0.008 | 1.00 | 2716 |
| Latitude_scaled | 0.088 | 0.060 | 0.116 | 1.00 | 1433 |
| Longitude_scaled | -0.016 | -0.044 | 0.011 | 1.00 | 1797 |
| ProtectionStatusProtected | -0.022 | -0.092 | 0.050 | 1.00 | 1977 |
| ProtectionStatusRamsarSPA | 0.062 | -0.009 | 0.134 | 1.00 | 1581 |
| Seasonal_tas_scaled:Latitude_scaled | -0.004 | -0.020 | 0.013 | 1.00 | 2153 |
| Seasonal_tas_scaled:Longitude_scaled | 0.021 | 0.005 | 0.038 | 1.00 | 2848 |
| Latitude_scaled:Longitude_scaled | 0.044 | 0.020 | 0.067 | 1.00 | 1882 |
| Seasonal_tas_scaled:ProtectionStatusProtected | 0.027 | -0.018 | 0.073 | 1.00 | 3481 |
| Seasonal_tas_scaled:ProtectionStatusRamsarSPA | 0.025 | -0.025 | 0.076 | 1.00 | 2937 |
| Latitude_scaled:ProtectionStatusProtected | 0.045 | -0.053 | 0.142 | 1.00 | 1896 |
| Latitude_scaled:ProtectionStatusRamsarSPA | 0.053 | -0.027 | 0.133 | 1.00 | 2043 |
| Longitude_scaled:ProtectionStatusProtected | 0.035 | -0.035 | 0.104 | 1.00 | 2045 |
| Longitude_scaled:ProtectionStatusRamsarSPA | 0.021 | -0.042 | 0.084 | 1.00 | 1984 |
| Seasonal_tas_scaled:Latitude_scaled:Longitude_scaled | -0.008 | -0.019 | 0.003 | 1.00 | 2412 |
| Seasonal_tas_scaled:Latitude_scaled:ProtectionStatusProtected | -0.034 | -0.090 | 0.021 | 1.00 | 3008 |
| Seasonal_tas_scaled:Latitude_scaled:ProtectionStatusRamsarSPA | -0.041 | -0.081 | 0.000 | 1.00 | 2271 |
| Seasonal_tas_scaled:Longitude_scaled:ProtectionStatusProtected | -0.017 | -0.060 | 0.027 | 1.00 | 2997 |
| Seasonal_tas_scaled:Longitude_scaled:ProtectionStatusRamsarSPA | -0.050 | -0.096 | -0.002 | 1.00 | 2704 |
| Latitude_scaled:Longitude_scaled:ProtectionStatusProtected | -0.054 | -0.129 | 0.016 | 1.00 | 2076 |
| Latitude_scaled:Longitude_scaled:ProtectionStatusRamsarSPA | 0.012 | -0.046 | 0.072 | 1.00 | 2206 |
| Seasonal_tas_scaled:Latitude_scaled:Longitude_scaled:ProtectionStatusProtected | 0.038 | 0.001 | 0.077 | 1.00 | 3247 |
| Seasonal_tas_scaled:Latitude_scaled:Longitude_scaled:ProtectionStatusRamsarSPA | -0.006 | -0.034 | 0.022 | 1.00 | 2451 |

**Table S9.** The coefficients from the functional richness and time model, including the median and lower and upper 95% credible intervals of the posterior distribution. Rhat close to one indicates convergence, and ESS is the effective sample size.

| Parameter | Median | Lower CI (95%) | Upper CI (95%) | Rhat | ESS |
| --- | --- | --- | --- | --- | --- |
| Intercept | 115.892 | 114.937 | 116.859 | 1.00 | 1204 |
| CentreYear | 1.260 | 1.178 | 1.336 | 1.00 | 2065 |
| Latitude_scaled | -0.804 | -1.657 | 0.054 | 1.00 | 1189 |
| Longitude_scaled | -3.287 | -4.109 | -2.465 | 1.00 | 1638 |
| ProtectionStatusProtected | -0.314 | -1.855 | 1.227 | 1.00 | 2736 |
| ProtectionStatusRamsarSPA | -0.453 | -2.023 | 1.136 | 1.00 | 3802 |
| CentreYear:Latitude_scaled | -0.185 | -0.266 | -0.105 | 1.00 | 2240 |
| CentreYear:Longitude_scaled | -0.016 | -0.093 | 0.063 | 1.00 | 2373 |
| Latitude_scaled:Longitude_scaled | -1.630 | -2.327 | -0.938 | 1.00 | 1605 |
| CentreYear:ProtectionStatusProtected | 0.159 | -0.039 | 0.362 | 1.00 | 2130 |
| CentreYear:ProtectionStatusRamsarSPA | -0.304 | -0.505 | -0.096 | 1.00 | 2999 |
| Latitude_scaled:ProtectionStatusProtected | -0.509 | -2.264 | 1.256 | 1.00 | 5076 |
| Latitude_scaled:ProtectionStatusRamsarSPA | -1.119 | -2.658 | 0.430 | 1.00 | 3675 |
| Longitude_scaled:ProtectionStatusProtected | -0.054 | -1.637 | 1.491 | 1.00 | 3162 |
| Longitude_scaled:ProtectionStatusRamsarSPA | -0.239 | -1.822 | 1.315 | 1.00 | 3574 |
| CentreYear:Latitude_scaled:Longitude_scaled | 0.047 | -0.017 | 0.112 | 1.00 | 2733 |
| CentreYear:Latitude_scaled:ProtectionStatusProtected | 0.191 | -0.100 | 0.481 | 1.00 | 2744 |
| CentreYear:Latitude_scaled:ProtectionStatusRamsarSPA | 0.080 | -0.137 | 0.289 | 1.00 | 2975 |
| CentreYear:Longitude_scaled:ProtectionStatusProtected | -0.154 | -0.353 | 0.046 | 1.00 | 2335 |
| CentreYear:Longitude_scaled:ProtectionStatusRamsarSPA | -0.004 | -0.213 | 0.200 | 1.00 | 3302 |
| Latitude_scaled:Longitude_scaled:ProtectionStatusProtected | 0.329 | -1.357 | 1.974 | 1.00 | 4288 |
| Latitude_scaled:Longitude_scaled:ProtectionStatusRamsarSPA | -1.192 | -2.542 | 0.160 | 1.00 | 3534 |
| CentreYear:Latitude_scaled:Longitude_scaled:ProtectionStatusProtected | 0.083 | -0.132 | 0.299 | 1.00 | 3227 |
| CentreYear:Latitude_scaled:Longitude_scaled:ProtectionStatusRamsarSPA | 0.051 | -0.116 | 0.213 | 1.00 | 4030 |

**Table S10.** The coefficients from the functional divergence and time model, including the median and lower and upper 95% credible intervals of the posterior distribution. Rhat close to one indicates convergence, and ESS is the effective sample size.

| Parameter | Median | Lower CI (95%) | Upper CI (95%) | Rhat | ESS |
| --- | --- | --- | --- | --- | --- |
| Intercept | 0.778 | 0.761 | 0.794 | 1.00 | 1311 |
| CentreYear | 0.005 | 0.004 | 0.006 | 1.00 | 5112 |
| Latitude_scaled | -0.041 | -0.058 | -0.025 | 1.00 | 1215 |
| Longitude_scaled | -0.040 | -0.055 | -0.025 | 1.00 | 1611 |
| ProtectionStatusProtected | -0.001 | -0.042 | 0.041 | 1.00 | 1883 |
| ProtectionStatusRamsarSPA | -0.058 | -0.100 | -0.014 | 1.00 | 1797 |
| CentreYear:Latitude_scaled | 0.002 | 0.001 | 0.003 | 1.00 | 5207 |
| CentreYear:Longitude_scaled | 0.000 | -0.001 | 0.001 | 1.00 | 6047 |
| Latitude_scaled:Longitude_scaled | -0.001 | -0.013 | 0.011 | 1.00 | 1923 |
| CentreYear:ProtectionStatusProtected | 0.000 | -0.002 | 0.003 | 1.00 | 5067 |
| CentreYear:ProtectionStatusRamsarSPA | -0.006 | -0.009 | -0.003 | 1.00 | 4981 |
| Latitude_scaled:ProtectionStatusProtected | 0.009 | -0.054 | 0.072 | 1.00 | 2275 |
| Latitude_scaled:ProtectionStatusRamsarSPA | 0.042 | 0.002 | 0.083 | 1.00 | 1893 |
| Longitude_scaled:ProtectionStatusProtected | 0.011 | -0.031 | 0.054 | 1.00 | 2199 |
| Longitude_scaled:ProtectionStatusRamsarSPA | 0.053 | 0.012 | 0.094 | 1.00 | 2077 |
| CentreYear:Latitude_scaled:Longitude_scaled | 0.000 | -0.001 | 0.001 | 1.00 | 6549 |
| CentreYear:Latitude_scaled:ProtectionStatusProtected | 0.000 | -0.004 | 0.004 | 1.00 | 5049 |
| CentreYear:Latitude_scaled:ProtectionStatusRamsarSPA | 0.000 | -0.003 | 0.003 | 1.00 | 6000 |
| CentreYear:Longitude_scaled:ProtectionStatusProtected | -0.001 | -0.004 | 0.002 | 1.00 | 4720 |
| CentreYear:Longitude_scaled:ProtectionStatusRamsarSPA | -0.001 | -0.004 | 0.002 | 1.00 | 6474 |
| Latitude_scaled:Longitude_scaled:ProtectionStatusProtected | 0.032 | -0.017 | 0.081 | 1.00 | 2739 |
| Latitude_scaled:Longitude_scaled:ProtectionStatusRamsarSPA | -0.007 | -0.038 | 0.024 | 1.00 | 2319 |
| CentreYear:Latitude_scaled:Longitude_scaled:ProtectionStatusProtected | 0.000 | -0.004 | 0.003 | 1.00 | 4786 |
| CentreYear:Latitude_scaled:Longitude_scaled:ProtectionStatusRamsarSPA | -0.001 | -0.004 | 0.001 | 1.00 | 5953 |

**Table S11.** The coefficients from the functional evenness and time model, including the median and lower and upper 95% credible intervals of the posterior distribution. Rhat close to one indicates convergence, and ESS is the effective sample size.

| Parameter | Median | Lower CI (95%) | Upper CI (95%) | Rhat | ESS |
| --- | --- | --- | --- | --- | --- |
| Intercept | -0.735 | -0.743 | -0.726 | 1.00 | 839 |
| CentreYear | -0.001 | -0.001 | 0.000 | 1.00 | 3263 |
| Latitude_scaled | 0.023 | 0.015 | 0.032 | 1.00 | 937 |
| Longitude_scaled | -0.051 | -0.060 | -0.044 | 1.00 | 1255 |
| ProtectionStatusProtected | 0.009 | -0.013 | 0.032 | 1.00 | 1098 |
| ProtectionStatusRamsarSPA | -0.039 | -0.062 | -0.015 | 1.00 | 1001 |
| CentreYear:Latitude_scaled | 0.000 | -0.001 | 0.000 | 1.00 | 3730 |
| CentreYear:Longitude_scaled | 0.000 | -0.001 | 0.000 | 1.00 | 3056 |
| Latitude_scaled:Longitude_scaled | -0.017 | -0.023 | -0.010 | 1.00 | 1517 |
| CentreYear:ProtectionStatusProtected | 0.001 | 0.000 | 0.003 | 1.00 | 3179 |
| CentreYear:ProtectionStatusRamsarSPA | 0.000 | -0.002 | 0.001 | 1.00 | 4391 |
| Latitude_scaled:ProtectionStatusProtected | 0.005 | -0.028 | 0.038 | 1.00 | 1383 |
| Latitude_scaled:ProtectionStatusRamsarSPA | -0.024 | -0.045 | -0.002 | 1.00 | 1317 |
| Longitude_scaled:ProtectionStatusProtected | -0.026 | -0.049 | -0.004 | 1.00 | 1357 |
| Longitude_scaled:ProtectionStatusRamsarSPA | 0.009 | -0.013 | 0.030 | 1.00 | 1248 |
| CentreYear:Latitude_scaled:Longitude_scaled | 0.000 | -0.001 | 0.000 | 1.00 | 4473 |
| CentreYear:Latitude_scaled:ProtectionStatusProtected | -0.001 | -0.003 | 0.001 | 1.00 | 3695 |
| CentreYear:Latitude_scaled:ProtectionStatusRamsarSPA | 0.001 | -0.001 | 0.002 | 1.00 | 4685 |
| CentreYear:Longitude_scaled:ProtectionStatusProtected | 0.002 | 0.000 | 0.003 | 1.00 | 3162 |
| CentreYear:Longitude_scaled:ProtectionStatusRamsarSPA | 0.000 | -0.001 | 0.002 | 1.00 | 4038 |
| Latitude_scaled:Longitude_scaled:ProtectionStatusProtected | 0.001 | -0.025 | 0.026 | 1.00 | 1763 |
| Latitude_scaled:Longitude_scaled:ProtectionStatusRamsarSPA | 0.019 | 0.003 | 0.036 | 1.00 | 1293 |
| CentreYear:Latitude_scaled:Longitude_scaled:ProtectionStatusProtected | -0.001 | -0.003 | 0.001 | 1.00 | 3312 |
| CentreYear:Latitude_scaled:Longitude_scaled:ProtectionStatusRamsarSPA | -0.001 | -0.002 | 0.000 | 1.00 | 4017 |

**Table S12.** The coefficients from the relative redundancy and time model, including the median and lower and upper 95% credible intervals of the posterior distribution. Rhat close to one indicates convergence, and ESS is the effective sample size.

| Parameter | Median | Lower CI (95%) | Upper CI (95%) | Rhat | ESS |
| --- | --- | --- | --- | --- | --- |
| Intercept | -1.797 | -1.824 | -1.772 | 1.00 | 1647 |
| CentreYear | -0.012 | -0.014 | -0.010 | 1.00 | 3353 |
| Latitude_scaled | 0.116 | 0.090 | 0.141 | 1.00 | 1743 |
| Longitude_scaled | 0.006 | -0.019 | 0.030 | 1.00 | 2366 |
| ProtectionStatusProtected | -0.003 | -0.073 | 0.067 | 1.00 | 1954 |
| ProtectionStatusRamsarSPA | 0.076 | 0.008 | 0.148 | 1.00 | 2161 |
| CentreYear:Latitude_scaled | 0.000 | -0.002 | 0.002 | 1.00 | 4517 |
| CentreYear:Longitude_scaled | 0.006 | 0.004 | 0.008 | 1.00 | 4164 |
| Latitude_scaled:Longitude_scaled | 0.049 | 0.029 | 0.069 | 1.00 | 2507 |
| CentreYear:ProtectionStatusProtected | -0.003 | -0.009 | 0.002 | 1.00 | 3287 |
| CentreYear:ProtectionStatusRamsarSPA | 0.006 | 0.000 | 0.011 | 1.00 | 4072 |
| Latitude_scaled:ProtectionStatusProtected | -0.051 | -0.156 | 0.055 | 1.00 | 1996 |
| Latitude_scaled:ProtectionStatusRamsarSPA | 0.008 | -0.061 | 0.076 | 1.00 | 2111 |
| Longitude_scaled:ProtectionStatusProtected | -0.043 | -0.117 | 0.031 | 1.00 | 1905 |
| Longitude_scaled:ProtectionStatusRamsarSPA | -0.004 | -0.075 | 0.065 | 1.00 | 2444 |
| CentreYear:Latitude_scaled:Longitude_scaled | -0.002 | -0.004 | 0.000 | 1.00 | 4487 |
| CentreYear:Latitude_scaled:ProtectionStatusProtected | 0.002 | -0.006 | 0.011 | 1.00 | 2611 |
| CentreYear:Latitude_scaled:ProtectionStatusRamsarSPA | -0.002 | -0.008 | 0.003 | 1.00 | 3578 |
| CentreYear:Longitude_scaled:ProtectionStatusProtected | 0.004 | -0.002 | 0.010 | 1.00 | 2352 |
| CentreYear:Longitude_scaled:ProtectionStatusRamsarSPA | -0.003 | -0.009 | 0.003 | 1.00 | 3555 |
| Latitude_scaled:Longitude_scaled:ProtectionStatusProtected | -0.033 | -0.118 | 0.054 | 1.00 | 2536 |
| Latitude_scaled:Longitude_scaled:ProtectionStatusRamsarSPA | 0.037 | -0.016 | 0.092 | 1.00 | 2312 |
| CentreYear:Latitude_scaled:Longitude_scaled:ProtectionStatusProtected | 0.003 | -0.004 | 0.010 | 1.00 | 2979 |
| CentreYear:Latitude_scaled:Longitude_scaled:ProtectionStatusRamsarSPA | 0.002 | -0.003 | 0.007 | 1.00 | 3517 |

## **References**

Aarif, K., Zouhar, J., Musilova, Z., Musil, P., Nefla, A., Muzaffar, S., et al. (2024). Bill length of non-breeding shorebirds influences the water depth preferences for foraging in the West Coast of India. *Ecol. Evol.*, 14, e70396.

Albaladejo-Robles, G., Böhm, M., Newbold, T. (2023). Species life-history strategies affect population responses to temperature and land-cover changes. *Glob. Chang. Biol.*, 29, 97–109.

Bregman, T.P., Sekercioglu, C.H., Tobias, J.A. (2014). Global patterns and predictors of bird species responses to forest fragmentation: Implications for ecosystem function and conservation. *Biol. Conserv.*, 169, 372–383.

Brown, J.H., Calder, W.A., Kodric-Brown, A. (1978). Correlates and consequences of body size in nectar-feeding birds. *Am. Zool.*, 18, 687–700.

Brown, J.H., Gillooly, J.F., Allen, A.P., Savage, V.M., West, G.B. (2004). Toward a metabolic theory of ecology. *Ecology*, 85, 1771–1789.

Burin, G., Kissling, W.D., Guimarães, P.R., Şekercioglu, C.H., Quental, T.B. (2016). Omnivory in birds is a macroevolutionary sink. *Nat. Commun.*, 7, 11250.

Capdevila, P., O’Brien, D., Marconi, V., Johnson, T.F., Freeman, R., McRae, L., et al. (2026). Halting predicted vertebrate declines requires tackling multiple drivers of biodiversity loss. *Sci. Adv.*, 12, eadx7973.

Cardillo, M., Mace, M.G., Jones, K.E., Bielby, J., Bininda-Edmonds, O.R., Sechrest, W., et al. (2005)*.* Multiple causes of high extinction risk in large mammal species. *Science*, 309, 1239–1241.

Claramunt, S., Derryberry, E.P., Remsen, J.V., Brumfield, R.T. (2012). High dispersal ability inhibits speciation in a continental radiation of passerine birds. *Proc. R. Soc. B.*,  279, 1567–1574.

Ducatez, S., Clavel, J., Lefebvre, L. (2015). Ecological generalism and behavioural innovation in birds: technical intelligence or the simple incorporation of new foods? *J. Anim. Ecol.*, 84, 79–89.

Duffy, J.E. (2002). Biodiversity and ecosystem function: The consumer connection. *Oikos*, 99, 201–219.

Fitzpatrick, J.W. (1985). Form, foraging behavior, and adaptive radiation in the Tyrannidae. *Ornithol. Monogr.*, 36, 447–470.

Gardner, J.L., Peters, A., Kearney, M., Joseph, L., Heinsohn, R. (2011). Declining body size: a third universal response to warming? *Trends Ecol. Evol.*, 26, 285–291.

Gelman, A., Goodrich, B., Gabry, J., Vehtari, A. (2019). R-squared for Bayesian Regression Models. *Am. Stat.*, 73, 307–309.

Goolsby, E.W., Bruggeman, J., Ané, C. (2017). Rphylopars: fast multivariate phylogenetic comparative methods for missing data and within-species variation. *Methods Ecol. Evol.*, 8, 22–27.

Habel, J.C., Tobias, J.A., Fischer, C. (2019). Movement ecology of Afrotropical birds: functional traits provide complementary insights to species identity. *Biotropica*, 51, 894–902.

Hackett, S.J., Kimball, R.T., Reddy, S., Bowie, R.C.K., Braun, E.L., Braun, M., et al. (2008). A phylogenomic study of birds reveals their evolutionary history. *Science*, 320, 1763–1768.

Hordley, L.A., Gillings, S., Petchey, O.L., Tobias, J.A., Oliver, T.H. (2021) Diversity of response and effect traits provides complementary information about avian community dynamics linked to ecological function. *Funct. Ecol.*, 35, 1938–1950.

Jetz, W., Thomas, G.H., Joy, J.B., Hartmann, K., Mooers, A.O. (2012). The global diversity of birds in space and time. *Nature,* 491, 444–448.

Johnson, T.F., Isaac, N.J.B., Paviolo, A., González-Suárez, M. (2021). Handling missing values in trait data. *Glob. Ecol. Biogoegr.*, 30, 51–62.

Keum, J., Sayol, F., Orgeret, F., Horswill, C. (2025). Does brain size matter? Linking cognitive and ecological traits to climate change vulnerability in seabirds. *Funct. Ecol.*, 39, 1061–1070.

Kisel, Y., Barraclogh T.G. (2010). Speciation has a spatial scale that depends on levels of gene flow. *Am. Nat.*, 175, 316–334.

Korpimäki, E., Hongisto, K., Masoero, G., Laaksonen, T. (2020). The difference between generalist and specialist: the effects of wide fluctuations in main food abundance on numbers and reproduction of two co-existing predators. *J. Avian Biol*., 51, e02508.

Larsen, T.H., Williams, N.M., Kremen, C. (2005). Extinction order and altered community structure rapidly disrupt ecosystem functioning. *Ecol. Lett.*, 8, 538–47.

Lefcheck, J.S., Duffy, J.E. (2015). Multitrophic functional diversity predicts ecosystem functioning in experimental assemblages of estuarine consumers. *Ecology*, 96, 2973–2983.

Li, Z., Zhang, H., Xu, Y., Wang, S. (2021) Composition of ‘fast-slow’ traits drives avian community stability over North America. *Funct. Ecol.*, 35, 2831–2840.

Linhart, R. C.,  Hamilton, D.J., Paquet, J.N., Monteiro, J.O.P., Ramires, G., Mobley, J.A. (2022). Movement and habitat use of non-breeding semipalmated sandpiper (*Calidris pusilla*) at the Banco dos Cajuais in Northeast Brazil. *Conserv. Sci. Pract.*, 4, e12683.

Lockwood, R., Swaddle, J.P., Rayner, J.M.V. (1998). Avian wingtip shape reconsidered: Wingtip shape indices and morphological adaptations to migration. *J. Avian Biol.*, 29, 273–292.

Mammola S., Cardoso P. (2020). Functional diversity metrics using kernel density n‐dimensional hypervolumes. *Methods Ecol. Evol.*, 11, 986–995.

Miles, D.B., Ricklefs, R.E. (1984). The Correlation Between Ecology and Morphology in Deciduous Forest Passerine Birds. *Ecology*, 65, 1629–1640.

Minias, P., Podlaszczuk, P. (2017) Longevity is associated with relative brain size in birds. *Ecol. Evol.*, 7, 3558-–566.

Newbold, T., Scharlemann, J.P.W., Butchart, S.H.M., Sekercioglu, C.H., Alkemade, R., Booth, H., et al. (2013). Ecological traits affect the response of tropical forest bird species to land-use intensity. *Proc. R. Soc. B. Biol. Sci.*, 280, 20122131.

Norazlimi, N. A., R. Ramli. (2015). The relationships between morphological characteristics and foraging behavior in four selected species of shorebirds and water birds utilizing tropical mudflats. *Sci. World J.*,  2015, 105296.

Ntiamoa-Baidu, Y., Piersma, T., Wiersma, P., Poot, M., Battley, P., Gordon, C. (1998). Water depth selection, daily feeding routines and diets of waterbirds in coastal lagoons in Ghana. *Ibis*, 140, 89–103.

Ohlert, T., Kimmel, K., Avolio, M., Chang, C., Forrestel, E., Gerstner, B.P., et al. (2024). The impact of trait number and correlation on functional diversity metrics in real-world ecosystems. *PLoS One*, 19, e0306342.

Penone, C., Davidson, A.D., Shoemaker, K.T., Di Marco, M., Rondinini, C., Brooks, T.M., et al. (2014). Imputation of missing data in life-history trait datasets: which approach performs the best? *Methods Ecol. Evol.*, 5, 961–970.

Pigot, A.L., Sheard, C., Miller, E.T., Bregman, T.P., Freeman, B.G., Roll, U., et al. (2020). Macroevolutionary convergence connects morphological form to ecological function in birds. *Nat. Ecol. Evol.*, 4, 230–239.

Pigot , A.L., Trisos, C.H., Tobias, J.A. (2016). Functional traits reveal the expansion and packing of ecological niche space underlying an elevational diversity gradient in passerine birds. *Proc. R. Soc. B.*, 283, 20152013.

Porter, W.P., Kearney, M. (2009). Size, shape, and the thermal niche of endotherms. *Proc. Natl. Acad. Sci. U.S.A.*, 106, 19666–19672.

Probst, C.M., Ralston, J., Bentley, I. (2022). Effects of climate on bill morphology within and across *Toxostoma* thrashers. *J. Avian Biol.*, 2022, e02871.

Promislow, D.E.L., Harvey, P.H. (1990). Living fast and dying young: A comparative analysis of life-history variation among mammals. *J. Zool.*, 220, 417–437.

Schoener, T.W. (1971). Large-billed insectivorous birds: a precipitous diversity gradient. *Condor*, 73, 154–161.

Sheard, C., Neate-Clegg, M.H.C., Alioravainen, N., Jones, S.E.I., Vincent, C. MacGregor, H.E.A., et al. (2020)*.* Ecological drivers of global gradients in avian dispersal inferred from wing morphology. *Nat. Commun.*, 11, 2463.

Soriano-Redondo, A., Gutierres, J.S., Hodgson, D., Bearhop, S. (2020) Migrant birds and mammals live faster than residents. *Nat. Commun.*, 11, 5719.

Stekhoven, D.J. (2022). missForest: Nonparametric Missing Value Imputation using Random Forest. R package version 1.5.

Stekhoven, D.J., Bühlmann, P. (2012). MissForest—Non-parametric missing value imputation for mixed-type data. *Bioinform.*, 28, 112–118.

Tobias, J.A., Sheard, C., Pigot, A.L., Devenish, A.J.M., Yang, J., Sayol, F., et al. (2022). AVONET: morphological, ecological and geographical data for all birds. *Ecol. Lett.*, 25, 581–597.

Travis, J.M.J., Delgado, M., Bocedi, G., Baguette, M., Barton, K., Bonte, D., et al. (2013). Dispersal and species’ responses to climate change. *Oikos*, 122, 1532–1540.

Viana, D.S., Gangoso, L., Bouten, W., Figuerola, J. (2016). Overseas seed dispersal by migratory birds. *Proc. R. Soc. B*., 283, 20152406.

Wilman, H., Belmaker, J., Simpson, J., de la Rosa, C., Rivadeneira, M.M., Jetz, W. (2014). EltonTraits 1.0: Species-level foraging attributes of the world’s birds and mammals. *Ecology*, 95, 2027–2027.

Yu, C., Zhang, R., Zhou, L., Cheng, L., Bao, Y., Song., Y. (2023). Morphological characteristics influence the spatial mixing patterns of shorebirds at Shengjin Lake. *Ecol. Evol.*, 13, e10054.

Zhang, S.-D., Ma, Z., Choi, C.-Y., Peng, H.-B., Melville, D.S., Zhao, T.T., et al. (2019). Morphological and digestive adjustments buffer performance: How staging shorebirds cope with severe food declines. *Ecol. Evol.*, 9, 3868–3878.

Zimova, M., Weeks, B.C., Willard, D.E., Giery, S.T., Jirinec, V.,  Burner, R.C., et al. (2023). Body size predicts the rate of contemporary morphological change in birds. *Proc. Natl. Acad. Sci. U.S.A.*, 120, e2206971120.

Zuur, A.F., Ieno, E.N., Elphick, C.S. (2010). A protocol for data exploration to avoid common statistical problems. *Methods Ecol. Evol.*, 1, 3–14.

Zwarts, L., Bijlsma, R.G., van der Kamp, J. (2023). Granivorous birds in the Sahel: Is seed supply limiting bird numbers? *Ardea*, 111, 283–304.
